# Early-Life Hydrogen Sulfide Signaling as a Target for Cardiovascular–Kidney–Metabolic Syndrome Reprogramming

**DOI:** 10.3390/antiox14091064

**Published:** 2025-08-29

**Authors:** Chien-Ning Hsu, Ying-Jui Lin, Chih-Yao Hou, Yu-Wei Chen, You-Lin Tain

**Affiliations:** 1 Department of Pharmacy, Kaohsiung Chang Gung Memorial Hospital, Kaohsiung 833, Taiwan; cnhsu@cgmh.org.tw; 2 Department of Pharmacy, Kaohsiung Municipal Ta-Tung Hospital, Kaohsiung 801, Taiwan; 3 School of Pharmacy, Kaohsiung Medical University, Kaohsiung 807, Taiwan; 4 Division of Critical Care, Department of Pediatrics, Kaohsiung Chang Gung Memorial Hospital, Chang Gung University College of Medicine, Kaohsiung 833, Taiwan; rayray@adm.cgmh.org.tw; 5 Department of Respiratory Therapy, Kaohsiung Chang Gung Memorial Hospital, College of Medicine, Chang Gung University, Kaohsiung 833, Taiwan; 6 Division of Cardiology, Department of Pediatrics, Kaohsiung Chang Gung Memorial Hospital, College of Medicine, Chang Gung University, Kaohsiung 833, Taiwan; 7 Department of Early Childhood Care and Education, Cheng Shiu University, Kaohsiung 833, Taiwan; 8 Department of Seafood Science, National Kaohsiung University of Science and Technology, Kaohsiung 811, Taiwan; chihyaohou@nkust.edu.tw; 9 Department of Food Science and Biotechnology, National Chung Hsing University, Taichung 402, Taiwan; d112043001@mail.nchu.edu.tw; 10 Department of Pediatrics, Kaohsiung Chang Gung Memorial Hospital, Kaohsiung 833, Taiwan; 11 Department of Pediatrics, Kaohsiung Municipal Ta-Tung Hospital, Kaohsiung 801, Taiwan; 12 College of Medicine, Chang Gung University, Taoyuan 333, Taiwan

**Keywords:** cardiovascular disease, hydrogen sulfide, kidney disease, sulfur, *N*-acetylcysteine, developmental origins of health and disease (DOHaD), developmental programming, metabolic syndrome

## Abstract

Hydrogen sulfide (H_2_S), once regarded solely as a toxic gas, is now recognized as a vital endogenous signaling molecule with important roles in both health and disease. Growing evidence supports the developmental origins of health and disease (DOHaD) framework, in which early-life disturbances in H_2_S signaling may drive the later development of cardiovascular–kidney–metabolic (CKM) syndrome—a condition that encompasses chronic kidney disease, obesity, diabetes, and cardiovascular disease. This review highlights the emerging importance of H_2_S in CKM programming and the potential of H_2_S-based interventions during gestation and lactation to prevent long-term adverse health outcomes in offspring. Findings from animal studies suggest that maternal supplementation with sulfur-containing amino acids, *N*-acetylcysteine, H_2_S donors, and related sulfur-containing biomolecules can attenuate CKM-related risks in progeny. Despite these advances, several critical areas remain underexplored, including the role of gut microbiota-derived H_2_S, the epigenetic mechanisms influenced by H_2_S during development, and the clinical translation of preclinical findings. Targeting H_2_S signaling offers a promising strategy for early-life prevention of CKM syndrome and may also hold broader potential for preventing other DOHaD-related chronic diseases.

## 1. Introduction

Hydrogen sulfide (H_2_S) is a colorless, toxic, corrosive, and flammable gas that naturally occurs in the environment at high concentrations [1,2]. However, at physiological levels (nanomolar to micromolar), H_2_S is endogenously generated and functions as a critical signaling molecule, contributing to cellular protection and the regulation of diverse biological processes [3,4,5].

H_2_S is part of a broader sulfur metabolism network that includes both enzymatic and non-enzymatic pathways [6,7]. This sulfur network comprises inorganic sulfur compounds (e.g., sulfite, sulfate, and thiosulfate), organic sulfur compounds (e.g., sulfur-containing amino acids and glutathione), and reactive sulfur species (RSS). The interactions between H_2_S and other sulfur species are essential for maintaining cellular redox balance and signaling [6,7].

Dietary sulfur is mainly derived from sulfur-containing amino acids and inorganic sulfate compounds. During gestation, adequate sulfate availability is crucial for fetal development, as the fetus relies heavily on maternal sulfate due to its limited capacity to synthesize it independently [8]. Maternal diet and sulfur metabolism are therefore key determinants influencing fetal growth and function through developmental programming [9]. Perturbations in early-life conditions can impair organ development, thereby increasing the risk of adult diseases—a concept known as the developmental origins of health and disease (DOHaD) [10].

Cardiovascular–kidney–metabolic (CKM) syndrome is an emerging global health challenge, driven by the interconnected epidemics of obesity, diabetes, chronic kidney disease (CKD), and cardiovascular disease (CVD) [11]. Although the term *CKM syndrome* was only introduced in 2023 [12], it is already estimated to affect nearly 90% of adults in the United States [13]. According to the DOHaD framework, early-life exposures can program the development of CKM syndrome in adulthood [14]. Importantly, these adverse programming effects may be reversible or delayed through early-life reprogramming strategies, offering new opportunities for CKM prevention [15].

Accumulating evidence links dysregulated sulfur metabolism to multiple pathological conditions—including metabolic syndrome [16], obesity [17], kidney disease [18], and CVD [19]—all central components of CKM syndrome. Conversely, targeting the H_2_S pathway has emerged as a promising reprogramming strategy to prevent DOHaD-related disorders [9]. Accordingly, this review summarizes the roles of H_2_S and related sulfur species in CKM programming and evaluates H_2_S-based interventions as potential strategies to prevent adult-onset CKM syndrome.

## 2. Material and Methods

A comprehensive literature search was conducted in Embase, MEDLINE, and the Cochrane Library to identify studies on hydrogen sulfide, CKM syndrome, and the DOHaD concept. The search encompassed both experimental and clinical studies published in English between January 2000 and April 2025. Keywords used in the strategy included, but were not limited to: “hydrogen sulfide,” “sulfur-containing amino acid,” “sulfur,” “sulfide,” “organosulfur compound,” “cysteine,” “obesity,” “diabetes,” “chronic kidney disease,” “metabolic syndrome,” “cardiovascular disease,” “hypertension,” “dyslipidemia,” “insulin resistance,” “hyperlipidemia,” “hyperglycemia,” “hepatic steatosis,” “atherosclerosis,” “heart failure,” “developmental programming,” “reprogramming,” “DOHaD,” “offspring,” “progeny,” “maternal,” “pregnancy,” and “lactation.” To ensure comprehensive coverage, the reference lists of eligible articles were also screened manually to identify additional relevant studies.

## 3. H_2_S and Sulfur Metabolism in Pregnancy

### 3.1. Biosynthesis of H_2_S

In 1989, more than two centuries after H_2_S was first recognized as a toxic gas [20], the discovery of its endogenous production in the brain revealed its potential involvement in physiological regulation [21]. H_2_S is now recognized as a gasotransmitter, alongside nitric oxide (NO) and carbon monoxide (CO), sharing several overlapping biochemical and signaling functions [22].

Cysteine and homocysteine serve as primary substrates for enzymatic H_2_S production. This occurs through the actions of cystathionine-γ-lyase (CSE; historically CGL) and cystathionine-β-synthase (CBS), both of which are localized primarily in the cytosol [23]. An alternative enzymatic pathway involves the metabolism of cysteine by 3-mercaptopyruvate sulfurtransferase (3-MST) and cysteine aminotransferase (CAT), which results in H_2_S generation. In this pathway, H_2_S is released from persulfidated 3-MST via the reducing activity of thioredoxin (Trx) or other cellular reductants. Furthermore, 3-MST produces H_2_S through a reaction that converts 3-mercaptopyruvate (3-MP) into pyruvate, with 3-MP supplied by CAT and D-amino acid oxidase (DAO). Notably, H_2_S can also be synthesized from D-cysteine by DAO in peroxisomes [24]. While 3-MST is found in both the cytoplasm and mitochondria, CBS and CSE are localized mainly to the cytosol.

Subsequent research has identified additional pathways contributing to H_2_S production. Methanethiol, for example, can be enzymatically converted into H_2_S via methanethiol oxidase [25]. More recently, mitochondrial cysteinyl-tRNA synthetase 2 was shown to catalyze the formation of cysteine persulfide (cysteine-SSH), representing a novel source of persulfidated proteins. These persulfidated proteins serve as a reservoir from which H_2_S can be released through the action of Trx or related reducing systems [26].

Beyond enzymatic mechanisms, H_2_S can also be synthesized through non-enzymatic pathways. These reactions involve diverse sulfur-containing intermediates, including thiosulfate, glutathione, glucose, inorganic sulfur compounds, and naturally occurring organic polysulfides (e.g., in garlic). Among these, thiosulfate—both a metabolite of H_2_S degradation and a central intermediate in sulfur cycling—was identified as an important precursor for non-enzymatic H_2_S generation, via a reduction reaction using pyruvate as an electron donor [27].

Subsequent studies revealed that glucose can contribute to H_2_S production, either through NADPH oxidase activity on phosphogluconate or through glycolytic pathways. Glucose may also react with sulfur-containing amino acids (cysteine, methionine, or homocysteine) to produce gaseous sulfur species, including H_2_S and methanethiol. In addition, H_2_S can arise from the direct reduction of glutathione or inorganic sulfur compounds, as well as from nucleophilic substitution reactions of organic polysulfides, which generate both H_2_S and hydropolysulfides (RSSH) [28]. RSSH, through S-persulfidation of cysteine residues, can modulate the activity, stability, and interactions of key proteins, thereby regulating pathways involved in vascular tone, inflammation, mitochondrial function, and epigenetic control [29,30,31]. Consequently, RSSH represents a vital component of H_2_S-mediated cytoprotection and signaling, with dysregulation potentially contributing to cardiovascular pathologies [32,33].

The gut microbiota represents another important source of H_2_S. Early studies identified sulfate-reducing bacteria (SRB), particularly *Desulfovibrio*—which constitutes ~66% of all SRB in the human colon—as major producers of H_2_S, generating it through oxidation of organic compounds coupled with sulfate reduction. Subsequent work demonstrated that other intestinal bacteria, including *Escherichia coli*, *Enterobacter*, *Corynebacterium*, *Klebsiella*, *Bacillus*, *Rhodococcus*, *Salmonella* and *Staphylococcus* species, can also produce H_2_S via sulfite reduction [34].

**Figure 1:**
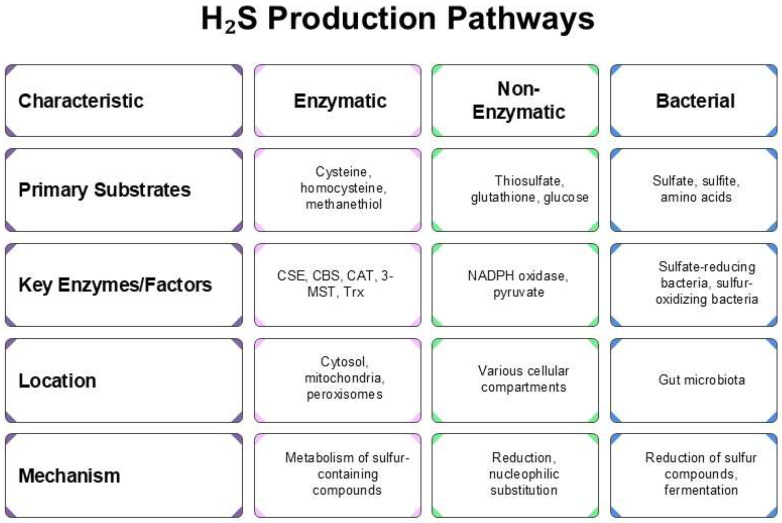
Schematic representation of enzymatic, nonenzymatic, and bacterial H_2_S synthesis pathways.

In contrast, sulfur-oxidizing bacteria (SOB) act as a counterbalance, lowering fecal H_2_S levels through sulfur oxidation. Moreover, gut-derived H_2_S can also arise from microbial fermentation of sulfur-containing amino acids. Once produced, a substantial portion of luminal H_2_S is detoxified by colonocytes through oxidation to thiosulfate [35]. Endogenous H_2_S originates from enzymatic, non-enzymatic, and bacterial pathways, which collectively and interactively regulate its physiological levels, as illustrated in Figure 1.

### 3.2. Molecular Targets of H_2_S

Hydrogen sulfide (H_2_S) exerts a wide range of physiological and pathophysiological effects through interactions with diverse molecular targets, including proteins, enzymes, ion channels, and signaling pathways. These effects arise through both direct chemical modifications of target molecules and indirect regulatory actions.

One of the earliest recognized mechanisms was S-sulfhydration, in which H_2_S modifies cysteine residues on proteins as a post-translational modification that alters protein structure and function [36]. For example, sulfhydration of the NF-κB p65 subunit attenuates inflammatory signaling, modification of Kelch-like ECH-associated protein 1 (Keap1) activates antioxidant defenses via the Nrf2 pathway, and modification of endothelial nitric oxide synthase (eNOS) enhances nitric oxide (NO) production and promotes vasodilation. Second, H_2_S also regulates membrane excitability and ion transport by modulating ion channels and transporters, including ATP-sensitive potassium (KATP) channels and Na^+^/K^+^-ATPase, thereby contributing to vascular tone and overall cellular homeostasis [37].

Third, H_2_S influences mitochondrial function, regulating energy production, redox balance, and ROS generation [38], and can modulate ferroptosis [39]. Ferroptosis, an iron-dependent, lipid peroxidation-driven form of cell death, contributes to the pathogenesis of kidney disease, CVD, and type 2 diabetes—key components of CKM syndrome [40,41,42]. Regulation of ferroptosis by H_2_S may thus represent a critical mechanism linking mitochondrial redox homeostasis to disease outcomes.

Fourth, H_2_S modulates epigenetic mechanisms [43], including the regulation of histone deacetylases (HDACs), DNA methyltransferases (DNMTs), and non-coding RNAs (ncRNAs), thereby influencing gene expression programs linked to inflammation and oxidative stress. Several cardiovascular pathologies—such as hypertension, congenital heart disease, heart failure, hyperhomocysteinemia, and atherosclerosis—are characterized by aberrant DNA hypermethylation [44]. A central mechanism involves S-persulfidation of cysteine residues, through which H_2_S regulates the activity of epigenetic enzymes (e.g., DNMTs and HDACs), ultimately shaping DNA methylation patterns [43]. For instance, H_2_S can inhibit HDAC6, restore CSE expression, improve endothelial function, and confer protection against CVDs [45]. In addition, ncRNAs function as downstream mediators of H_2_S signaling or as regulators of H_2_S-generating enzymes, thereby modulating endogenous H_2_S bioavailability [46]. Among ncRNAs, microRNAs (miRNAs) play a particularly important role. Notably, H_2_S interacts with miR-21: while H_2_S treatment downregulates miR-21, miR-21 upregulation suppresses CSE expression [47,48]. Treatment with H_2_S donors reduces miR-21 levels, thereby preventing cardiomyocyte hypertrophy and kidney injury [47,49]. Finally, H_2_S acts on immune cells and inflammatory mediators, suppressing the production of pro-inflammatory cytokines and exerting broad anti-inflammatory effects [50].

### 3.3. Sulfur Metabolism in Pregnancy and Its Impact on Fetal Development

Sulfur, an essential element, is the third most abundant mineral in the human body. The diet provides a wide range of both inorganic and organic sulfur-containing compounds. Dietary sulfur is derived from multiple sources, including inorganic forms like sulfate (SO_4_^2−^) and sulfite (SO_3_^2−^), which are commonly present in drinking water and processed foods. Protein-rich foods, especially meat, supply sulfur through amino acids such as methionine and cysteine, while vegetables—notably garlic and onions—provide additional sulfur through diverse organosulfur compounds [51]. Maternal dietary intake of these sulfur sources is increasingly recognized as a key determinant of long-term health outcomes in offspring [52]. As a product of sulfur metabolism, H_2_S, along with other sulfur-related metabolites, plays important physiological roles during normal pregnancy, particularly in supporting fetal growth and development [53,54].

#### 3.3.1. Sulfur-Containing Amino Acids in Pregnancy

During pregnancy, amino acids are essential for fetal development, with increased needs met through diet and maternal protein turnover. Among these, sulfur-containing amino acids—including methionine, cysteine, taurine, and homocysteine—play critical roles. Methionine and cysteine together account for approximately 4% of maternal proteins [55]. Methionine is especially critical, serving not only as a substrate for protein synthesis but also as a key component of one-carbon metabolism, which underpins DNA synthesis and epigenetic regulation [56]. Inadequate methionine intake has been associated with fetal growth restriction [57,58], while excessive intake may disturb the balance of other amino acids, such as glycine and serine [58].

Research has further shown that, during early gestation, increased maternal transsulfuration enhances the supply of cysteine and glutathione to the fetus, indicating a metabolic shift in methionine utilization [58]. Cysteine, a precursor of glutathione and hydrogen sulfide (H_2_S), is crucial for fetal antioxidant defense and vascular development [59,60]. Lower maternal plasma cysteine levels in late pregnancy reflect increased fetal demand [61].

**Figure 2:**
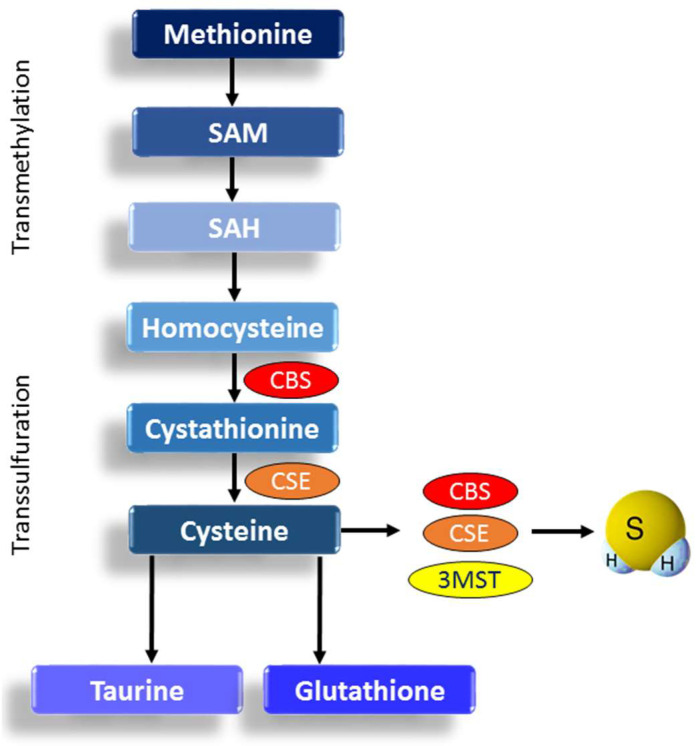
Schematic representation of the transmethylation and transsulfuration pathways, highlighting their roles in sulfur-containing amino acid metabolism and H_2_S production. SAM = S-adenosylmethionine (SAM); SAH = S-adenosylhomocysteine; CBS = cystathionine-β-synthase; CSE = cystathionine-γ-lyase; 3MST = 3-mercaptopyruvate sulfurtransferase.

Homocysteine, a methionine metabolite, is associated with adverse pregnancy outcomes when elevated. Interestingly, homocysteine levels typically decrease during healthy pregnancies, although the underlying mechanisms remain unclear [58,62]. Taurine, another sulfur-containing metabolite derived from cysteine, also plays a key role in fetal growth. Maternal taurine deficiency has been associated with low birth weight and increased risk of disease in later life [63,64]. The transmethylation and transsulfuration pathways involved in sulfur-containing amino acid metabolism and H_2_S production are illustrated in Figure 2.

#### 3.3.2. Sulfate

Beyond sulfur-containing amino acids, the human diet provides sulfur through a variety of other sources, including inorganic molecules like sulfate and sulfite, as well as naturally occurring organic sulfur compounds found in garlic, onions, and cruciferous vegetables. Sulfate is particularly abundant, being widely distributed in both food and drinking water. In the gastrointestinal tract, certain microbes—notably sulfate-reducing bacteria (SRB)—utilize sulfate as a terminal electron acceptor, generating hydrogen sulfide (H_2_S) as a metabolic byproduct [65,66].

Research indicates that sulfate plays an essential role in fetal development, with maternal sulfate insufficiency linked to impaired fetal growth [67]. During pregnancy, maternal plasma sulfate concentrations increase significantly, peaking in late gestation at nearly twice the levels observed in nonpregnant women [68]. This rise is largely attributed to enhanced renal reabsorption, driven by increased expression of the sodium-dependent sulfate transporter SLC13A1 in the maternal kidneys [69]. Sulfate is also actively transported across the placenta, where it supports sulfonation reactions vital for fetal tissue development and structural integrity [68].

#### 3.3.3. Organosulfur Compounds

Organosulfur compounds, found abundantly in Allium (e.g., garlic, onion) and Brassica (e.g., broccoli, cabbage) vegetables, support cellular metabolism and protect against oxidative stress [70]. These compounds, which include sulfoxides, sulfides, and glucosinolate derivatives, contain sulfur bonded to carbon or cyanate groups. In Allium species, key bioactives include allicin, S-allyl cysteine, and various sulfides, while Brassica vegetables are rich in glucosinolates, precursors to isothiocyanates [71]. Emerging evidence suggests potential benefits during pregnancy: one prospective cohort study reported that maternal garlic intake was associated with a reduced risk of spontaneous preterm birth [72]. Nevertheless, the optimal and safe intake levels of organosulfur compounds during pregnancy and lactation remain uncertain and warrant further investigation [73].

#### 3.3.4. H_2_S

H_2_S plays a vital role in pregnancy by promoting vasodilation of uterine and umbilical vessels [74], exerting tocolytic effects [75], and maintaining fetal membrane integrity [76]. It can also prolong labor duration and reduce uterine contraction frequency, supporting a smoother delivery process [77]. Dysregulated H_2_S signaling has been associated with complications such as embryonic resorption, ectopic pregnancy, and preeclampsia [78,79]. Additionally, by helping regulate homocysteine levels, H_2_S may reduce the risk of miscarriage, fetal abnormalities, and preeclampsia [80,81].

## 4. H_2_S Signaling and Cardiovascular–Kidney–Metabolic Health

CKM syndrome is categorized into four progressive stages (1–4), reflecting increasing severity and clinical complexity [12]. Unlike traditional metabolic syndrome, which focuses on obesity, hypertension, and dyslipidemia, CKM syndrome integrates metabolic, kidney, and cardiovascular components into a unified framework [12]. This concept recognizes the bidirectional interactions between early kidney dysfunction, metabolic disturbances, and subclinical cardiovascular changes, offering a more comprehensive understanding of disease progression and risk than conventional metabolic syndrome or isolated CKD. Stage 1 is marked by metabolic risk factors such as obesity, hypertension, or dyslipidemia without overt disease. Stage 2 involves the onset of CKD or established metabolic disorders like type 2 diabetes or non-alcoholic fatty liver disease (NAFLD). Stage 3 is characterized by subclinical cardiovascular disease, often without apparent symptoms. Stage 4 represents advanced CKD and clinical cardiovascular disease, carrying the highest risk of adverse outcomes. While the precise role of H_2_S in CKM syndrome remains to be fully defined, emerging evidence suggests it is a key mediator involved in the pathophysiology of several components of the syndrome.

### 4.1. Obesity and Diabetes

The World Health Organization (WHO) defines obesity as both a health urgency and a social emergency, noting that more than one-third of the global population is overweight, with over 13% classified as obese [82]. In obesity, adipocytes transition from healthy energy regulators to dysfunctional, pro-inflammatory cells that drive metabolic disease development [83]. Their secretory activity, interaction with immune cells, and impaired lipid handling all contribute to the pathogenesis of obesity-related complications such as type 2 diabetes, NAFLD, and CVD.

Emerging evidence highlights hydrogen sulfide (H_2_S) as a critical regulator of lipid and energy metabolism. At physiological levels, H_2_S promotes adipogenesis, lipolysis, and adipose tissue browning, enhancing energy expenditure, supporting glucose homeostasis, and improving insulin signaling [84,85]. It also exerts anti-inflammatory and antioxidant effects, helping mitigate chronic low-grade inflammation associated with metabolic disorders. Conversely, dysregulation of H_2_S production has been associated with adipose tissue dysfunction, impaired pancreatic β-cell function, and insulin resistance, thereby contributing to the onset and progression of obesity and type 2 diabetes [86].

### 4.2. Dyslipidemia and NAFLD

In the liver, both CBS and CSE knockout (KO) mouse models, as well as studies using exogenous H_2_S donors, have shown that H_2_S plays a key regulatory role in metabolic pathways such as gluconeogenesis, glucose utilization, glycogen synthesis, and triglyceride (TG) metabolism [87]. CBS KO models underscore the importance of CBS in hepatic lipid homeostasis; elevated homocysteine due to CBS deficiency disrupts lipid regulation by activating the unfolded protein response, increasing HMG-CoA reductase expression and cholesterol synthesis [88], and enhancing LDL receptor–mediated cholesterol uptake [89]. These molecular disturbances drive the accumulation of fatty acids and lipid intermediates in hepatocytes, thereby promoting the onset of dyslipidemia and non-alcoholic fatty liver disease (NAFLD).

Multiple studies have reported impaired hepatic H_2_S production in animal models of NAFLD [90,91], suggesting a link between reduced H_2_S bioavailability and disease progression. Therapeutically, H_2_S supplementation has shown promise in reversing NAFLD-related pathologies [92,93]. In rats fed a choline- and methionine-deficient diet, hepatic H_2_S levels declined alongside the development of steatosis and inflammation [90]; NaHS supplementation restored H_2_S levels, reduced mitochondrial ROS, and improved liver pathology. Similarly, in high-fat diet fed mice, NaHS treatment lowered hepatic lipid content, oxidative stress, and lipogenic enzyme expression while enhancing fatty acid oxidation [92], underscoring the protective role of H_2_S in dyslipidemia and NAFLD.

### 4.3. Kidney Disease and Hypertension

H_2_S has multiple roles in renal physiology, including regulation of renal blood flow [94], promoting natriuresis via reduction of Na^+^/K^+^-ATPase activity [95], modulation of glomerular filtration rate [96], reduce renin release [97], and regulation of BP [98]. Additially, H_2_S interacts with the renin-angiotensin system [99], NO pathway [100], and oxidative stress [101] pathways, influencing overall kidney function and systemic homeostasis.

Conversely, dysregulated H_2_S signaling contributes to the development of kidney disease and hypertension [102]. In animal models such as spontaneously hypertensive rats (SHR) and dexamethasone-induced hypertension, H_2_S deficiency precedes the onset of hypertension [103,104], while supplementation with exogenous H_2_S donors like NaHS confers protective effects [105]. Similar findings are observed in other hypertensive models, including renovascular hypertension [106], NO-deficient rats [107], and salt-sensitive rats [108]. Genetic deletion of the H_2_S-producing enzyme CSE also leads to reduced H_2_S levels and elevated BP, though results may vary by genetic background [109,110].

Beyond hypertension, H_2_S deficiency is linked to various kidney diseases, including ischemia/reperfusion injury, diabetic and hypertensive nephropathy, obstructive nephropathy, and CKD in the 5/6 nephrectomy model [111,112]. In contrast, therapeutic modulation of H_2_S (e.g., H_2_S donors or enzyme modulators) is being explored in renal protection and antihypertensive strategies [113].

### 4.4. Cardiovascular Disease

At physiological levels, H_2_S has a vital role in maintaining endothelial function and cardiovascular homeostasis. It exhibits potent cardioprotective properties, primarily by mitigating oxidative stress and inflammation within the cardiovascular system [19,114,115]. Deficiencies in H_2_S production or signaling are associated with various CVD, including atherosclerosis, myocardial infarction, heart failure, and cardiac hypertrophy [116,117]. Clinical studies consistently report significantly lower plasma H_2_S levels in patients with coronary artery disease, unstable angina, or heart failure [117]. Experimental evidence from preclinical models demonstrates that both endogenous H_2_S and exogenous H_2_S donors confer cardioprotection by reducing infarct size, enhancing cardiac function, promoting angiogenesis, and suppressing oxidative stress, inflammation, and apoptosis [118,119].

These effects are mediated through multiple mechanisms, such as activation of the AKT1–VEGF–NO signaling pathway [120], protein sulfhydration [36], epigenetic modulation [121], and regulation of mitochondrial function [38], and antioxidant effects [38]. Moreover, H_2_S interacts with NO and other reactive sulfur species, highlighting its complex and integrated role in cardiovascular physiology and the pathogenesis of CVD [122].

### 4.5. H_2_S Catabolism in CKM Syndrome

Hydrogen sulfide (H_2_S) catabolism is primarily mediated by the mitochondrial sulfide oxidation pathway [23], involving sulfide: quinone oxidoreductase (SQOR), persulfide dioxygenase (ETHE1), and thiosulfate sulfurtransferase (TST) [123,124,125]. This degradation process regulates H_2_S levels and maintains sulfur homeostasis. Dysregulated H_2_S catabolism perturbs sulfur homeostasis by causing either excessive H_2_S accumulation or depletion. Impaired mitochondrial sulfide oxidation—through dysfunction of SQOR, ETHE1, or TST—can elevate H_2_S to toxic levels, inhibiting cytochrome c oxidase, impairing oxidative phosphorylation, and driving mitochondrial dysfunction [126,127]. Conversely, accelerated clearance reduces H_2_S bioavailability, thereby diminishing its vasodilatory, antioxidant, and anti-inflammatory actions [128,129]. Such imbalances are increasingly implicated in CKM syndromes: in the kidney, they may exacerbate oxidative stress and tubular injury [130]; in the cardiovascular system, they promote endothelial dysfunction and vascular stiffness [131]; and in metabolic tissues, they interfere with insulin signaling and energy metabolism [132]. Together, these processes suggest that defective H_2_S catabolism contributes to the intertwined pathophysiology of CKM syndrome.

## 5. Role of H_2_S in Developmental Programming of CKM Syndrome

As CKMS is a newly defined multisystem disorder, no single animal model currently replicates all features of the human condition. In particular, the mechanisms underlying the developmental programming of CKM syndrome—induced during the early stages of life—may differ fundamentally from those driving the established CKM syndrome that arises in adulthood.

To date, various animal models exposed to distinct environmental insults have been developed to investigate specific components of CKM syndrome, including hypertension [133,134], kidney disease [135,136], metabolic syndrome [137,138], and cardiovascular disease [139]. Given the multi-organ dysfunction inherent to CKM syndrome, several models have successfully recapitulated multiple components of the syndrome in adult offspring. These models, as reviewed in prior studies [14], have been widely used to explore CKM syndrome from a developmental origin perspective. Notably, several of these models are directly associated with H_2_S signaling in CKM programming and will be discussed in greater detail below.

### 5.1. Maternal Nutritional Imbalance

Both excessive and insufficient intake of specific nutrients during gestation and lactation can trigger features of CKM syndrome with developmental origins in animal models. These include models based on low-calorie diets [140,141], low-protein diets [142,143], litter size reduction (to induce overnutrition) [144,145], as well as high-fat [146,147] and high-fructose diets [148,149].

Low-calorie and low-protein diet models have been widely utilized to investigate the mechanisms of nutritional programming, simulating conditions of human famine [150]. Because sulfate and sulfur-containing amino acids are essential for fetal development, their deficiency has been directly linked to impaired fetal growth [9,67]. Experimental evidence indicates that restricting either total caloric intake or dietary protein during early development predisposes offspring to a spectrum of cardiometabolic and renal disorders in adulthood, including excessive weight gain, impaired glucose metabolism, elevated blood pressure, cardiovascular dysfunction, and renal impairment [14,140,141,142,143].

Conversely, maternal overnutrition also contributes significantly to adverse offspring outcomes. Litter size reduction during lactation is an established method to induce overfeeding, promote accelerated neonatal growth, and trigger early-onset overweight or obesity in rodents [151]. Additionally, maternal diets high in fat or fructose have been extensively used to model developmental origins of CKM syndrome, resulting in offspring phenotypes characterized by obesity, insulin resistance, hypertension, and dyslipidemia [146,147,148,149,152,153].

Importantly, excessive intake of fat or fructose has been linked to disruption of the H_2_S-generating system [154,155]. However, only a limited number of studies have directly examined this relationship in DOHaD-related research; one study reported reduced H_2_S synthesis in the liver and adipose tissue of offspring exposed to a maternal high-fructose diet [156].

### 5.2. Maternal Illness

Gestational diabetes mellitus (GDM) is the most common pregnancy complication and is associated not only with maternal health issues but also with long-term adverse outcomes in offspring, as demonstrated in both human and animal studies [157,158]. Emerging evidence implicates deficient placental H_2_S synthesis in the pathogenesis of GDM [159]. Experimental models of maternal diabetes indicate that elevated maternal blood glucose during pregnancy programs long-term metabolic and cardiovascular disturbances in offspring, including increased susceptibility to obesity, impaired insulin sensitivity, dyslipidemia, hypertension, diabetes, and renal dysfunction in later life [160,161,162,163,164].

In addition to maternal diabetes, other maternal insults—such as uremia [165], uteroplacental insufficiency [166], hypoxia [167], hypoprolactinemia [168], and inflammation [169]—have also been associated with various features of CKM syndrome. In a maternal CKD model, adult offspring born to uremic dams exhibited reduced renal H_2_S synthesis [165]. Similarly, uteroplacental insufficiency downregulates placental CSE expression, which can lead to adverse fetal outcomes, although its long-term effects on CKM-related phenotypes in offspring remain to be fully elucidated [170].

### 5.3. Medication Use

Certain medications used during pregnancy and lactation have been shown to exert programming effects on offspring CKM outcomes. For instance, lactational exposure to metformin may induce long-term metabolic alterations in offspring [171]. Several drugs administered during pregnancy—such as cyclosporine A, nonsteroidal anti-inflammatory drugs, and gentamicin—have been demonstrated in animal models to impair nephrogenesis, thereby increasing the risk of hypertension and kidney disease in adult offspring [172,173,174]. While most of these agents affect specific organs, they do not comprehensively impact all components of the CKM spectrum. In contrast, in utero exposure to synthetic glucocorticoids has been shown to induce a broad constellation of CKM-related conditions, including obesity, insulin resistance, hypertension, and kidney disease in adult offspring [175,176,177]. This widespread effect is attributed to glucocorticoids’ regulation of the hypothalamic–pituitary–adrenal (HPA) axis, which orchestrates the function of multiple organ systems, including the liver, kidneys, and endocrine organs. Consequently, glucocorticoid-induced programming leads to persistent, organ-specific changes in gene expression that collectively contribute to CKM syndrome [178].

In dexamethasone-treated rats, hypertension has been linked to reduced expression of CBS and CSE, leading to decreased vascular H_2_S production [179]. Another study demonstrated that dexamethasone inhibits lipopolysaccharide-induced H_2_S biosynthesis both in vitro and in vivo [180], suggesting an interaction between glucocorticoids and the H_2_S signaling pathway. However, whether perinatal glucocorticoid exposure programs CKM syndrome in offspring specifically through H_2_S-dependent mechanisms remains to be fully elucidated.

Notably, over 100 FDA-approved drugs contain sulfur in various chemical forms, including thiols, sulfonamides, sulfoxides, sulfates, and thioethers [181,182]. These include ACE inhibitors, diuretics, sulfonylureas, proton pump inhibitors, antiepileptics, and D-penicillamine, among others. Some of these sulfur-containing drugs may interact with the H_2_S signaling pathway, particularly in cardiovascular, renal, and metabolic systems. As many of these agents are prescribed during pregnancy, their potential impact on H_2_S signaling and subsequent programming of offspring CKM outcomes warrants further investigation.

### 5.4. Chemical Exposure

Environmental chemicals can act as endocrine-disrupting chemicals (EDCs), not only increase the risk of pregnancy and fetal complications but also induce cross-generational effects through epigenetic mechanisms [183,184,185].

Animal studies have demonstrated that prenatal exposure to environmental chemicals such as 2,3,7,8-tetrachlorodibenzo-p-dioxin (TCDD) [186,187,188], bisphenol A (BPA) [189,190,191], and di-n-butyl phthalate (DEHP) [192,193,194,195] is associated with an increased risk of developing a spectrum of cardiometabolic and renal disorders in adult offspring, including obesity, high BP, insulin resistance, CKD, and CVD.

Emerging evidence suggests that EDCs can interfere with H_2_S signaling pathways by increasing oxidative stress and downregulating key H_2_S-producing enzymes [196]. Given that both EDC exposure and H_2_S deficiency are associated with obesity, insulin resistance, hypertension, and kidney disease, H_2_S likely represents a convergent mechanism underlying EDC-induced CKM programming. Further research is needed to determine whether restoring H_2_S signaling can mitigate the long-term adverse health effects of early-life EDC exposure.

It is also noteworthy that most animal models to date primarily involve rats and tend to focus on isolated components of CKM syndrome, rather than addressing the syndrome comprehensively. To advance DOHaD research, future studies should incorporate long-term follow-ups across diverse animal species to validate these findings. Such efforts are crucial for elucidating the role of H_2_S signaling and deepening our understanding of the mechanisms underlying CKM programming.

## 6. H_2_S-Based Reprogramming Interventions Against CKM Syndrome

The utilization of H_2_S-based therapy has been proven to yield benefits in many diseases, including a variety of CKM conditions [19,85,94,98,102,118]. Still, little attention has been paid to understanding perinatal H_2_S-based interventions for the prevention of offspring CKM syndrome [9,122]. Early intervention, even prior to the disease appearing, is key to preventing the development of adult disease, namely reprogramming [15,197]. Studies documenting H_2_S-based interventions in animal models for CKM reprogramming are summarized in Table 1, restricting interventions during pregnancy and lactation periods [163,198,199,200,201,202,203,204,205,206,207,208,209,210,211,212,213,214,215].

Table 1 highlights that rats are the predominant animal model used in studies investigating the developmental programming effects of H_2_S-based interventions, with mice being the second most common. A wide range of maternal insults-induced programming models have been employed to explore reprogramming potential, including those simulating CKD during pregnancy [198,212,213,215], gestational diabetes [199], excessive maternal sugar intake [201], cafeteria-style diets [200], genetic predisposition to hypertension [163,202,206], prenatal glucocorticoid exposure followed by postnatal high-fat diets [203], NO deficiency induced by L-NAME [204], suramin treatment during pregnancy [205], nicotine exposure in utero [207], maternal high-fat intake [208,209,214], and renovascular hypertension [210,211].

Among the offspring outcomes studied, hypertension is the most frequently reported, followed by kidney disease, fatty liver, lipid abnormalities, obesity, and impaired glucose metabolism. H_2_S-related reprogramming strategies include supplementation with sulfur-containing amino acids [163,198,199,200,201,202], administration of *N*-acetylcysteine (NAC) [203,204,205,206,207,208,209], application of H_2_S-releasing compounds [210,211,212], and treatment with naturally occurring sulfur-containing biomolecules [213,214,215]. Preclinical studies demonstrate that these interventions can effectively ameliorate disease phenotypes in rat offspring aged 8–32 weeks, corresponding to adolescence through young adulthood in humans [216].

### 6.1. Sulfur-Containing Amino Acids

L-cysteine and D-cysteine are both substrates for H_2_S production [24]. However, in the kidneys, the D-cysteine pathway exhibits approximately 80-fold higher H_2_S-producing activity compared to the L-cysteine pathway [217]. In a maternal CKD rat model, perinatal L- and D-cysteine supplementation showed different effects on offspring hypertension and kidney disease. L-cysteine boosted renal H_2_S-producing enzymes CBS and CSE, increased H_2_S release, and raised plasma H_2_S and thiosulfate. D-cysteine mainly restored plasma thiosulfate reduced by CKD, with little impact on renal enzyme expression [198].

Among sulfur-containing amino acids, taurine is the most extensively studied in the context of CKM reprogramming. Perinatal taurine supplementation has been shown to protect adult rat offspring against a range of CKM outcomes, including liver steatosis [199], obesity, dyslipidemia [200], hypertension [163,201,202], and kidney disease [201]. By contrast, high-methionine diets in animal models are linked to oxidative stress, hyperhomocysteinemia, and vascular dysfunction, all recognized risk factors for CKM syndrome [218,219]. Although methionine may influence CKM risk via epigenetic and metabolic pathways, current evidence is insufficient to recommend methionine modulation—particularly restriction—as a viable reprogramming strategy for CKM syndrome.

### 6.2. N-Acetylcysteine

NAC, a prodrug of L-cysteine, plays an indirect yet potentially significant role in H_2_S signaling, primarily by serving as a precursor to cysteine and acting as an antioxidant. Evidence from developmental programming studies indicates that perinatal NAC administration can prevent offspring hypertension induced by diverse maternal insults, including antenatal dexamethasone exposure combined with a post-weaning high-fat diet [203], maternal NO deficiency [204], suramin administration [205], hypertension [206], and nicotine exposure [207]. In the maternal NO-deficiency model, the protective effects of NAC on offspring BP were associated with upregulation of CBS and CSE, along with enhanced renal H_2_S production [204]. Furthermore, NAC has also been shown to ameliorate high-fat diet-induced glucose intolerance, dyslipidemia, and hepatic steatosis in offspring [208,209].

### 6.3. H_2_S Donors

Inorganic sulfide salts, such as sodium hydrosulfide (NaHS), are among the most commonly used exogenous H_2_S donors [219,220]. NaHS administration during pregnancy and lactation has been shown to prevent the development of hypertension in offspring within a maternal renovascular hypertensive model [210,211]. NaHS administration increased methylation of the angiotensin II receptor 1 (AT1R) gene, resulting in reduced transcription, lower BP, and improved cardiovascular homeostasis [210]. However, because inorganic sulfide salts release free H_2_S rapidly and at supraphysiological concentrations, organic slow-releasing H_2_S donors have been developed to overcome this limitation [221].

One of the earliest and most studied slow-releasing H_2_S donors is GYY4137 [221]. Although GYY4137 has demonstrated protective effects against hypertension in models involving CSE inhibition and L-NAME-treated SHRs [222,223], its role has not yet been evaluated in models of CKM programming.

Sodium thiosulfate (STS), unlike traditional H_2_S donors such as NaHS, does not spontaneously release H_2_S. Instead, it can be enzymatically converted back into H_2_S or other bioactive sulfur species. Growing evidence supports the therapeutic potential of STS in kidney disease [224,225]. Consistent with prior findings, our recent work demonstrated that STS can generate H_2_S and prevent hypertension in offspring exposed to maternal chronic kidney disease [226].

### 6.4. Sulfur-Containing Biomolecule

In addition to synthetic H_2_S donors, increasing attention has been directed toward natural sources of H_2_S, particularly organosulfur compounds. These include polysulfides derived from Allium species—such as diallyl disulfide and diallyl trisulfide—as well as glucosinolate-derived isothiocyanates [226].

Garlic, a rich source of organic polysulfides, has demonstrated potential benefits across multiple components of the CKM syndrome, including CVD, obesity, diabetes, dyslipidemia, and kidney disease [227,228,229,230]. Perinatal supplementation with garlic oil during gestation and lactation protects offspring from maternal CKD-induced hypertension at 12 weeks of age, an effect associated with elevated plasma H_2_S levels and increased renal expression of 3-MST protein [213]. In a separate maternal high-fat diet model, perinatal garlic oil supplementation prevented the development of hypertension in 16-week-old offspring, likely via enhanced renal H_2_S-releasing activity and upregulation of 3-MST mRNA expression [214].

Chondroitin sulfate, a sulfated glycosaminoglycan, has been reported to possess anti-inflammatory, antioxidant, anti-obesity, anti-cancer, and prebiotic properties [231]. As a sulfur-containing prebiotic [232,233], maternal supplementation with chondroitin sulfate protected offspring from maternal CKD-induced hypertension, which was associated with increased renal mRNA and protein expression of 3MST.

### 6.5. Others

The impact of gut-derived H_2_S in CKM programming remains largely unexplored, despite the fact that the intestinal microbiota represents the body’s primary source of H_2_S. In the gut, SRB and SOB work in tandem to regulate local H_2_S levels [234]. While physiological concentrations of H_2_S may support gut homeostasis, elevated levels are toxic to intestinal epithelial cells and have been implicated in gastrointestinal disorders. Interventions targeting SRB activity have been investigated as a means to limit inflammation-related H_2_S overproduction in the gut [235]. Given the increasing recognition of the gut–organ axis in systemic disease, future studies are warranted to determine whether gut microbiota-targeted interventions—including probiotics, prebiotics, and postbiotics [236,237,238]—can modulate microbial H_2_S production and thereby offer a novel avenue for reprogramming CKM-related conditions.

In addition to microbial sources, endogenous H_2_S signaling is influenced by several commonly prescribed medications, including aspirin, amlodipine, atorvastatin, carvedilol, testosterone, digoxin, cimetidine, metformin, paracetamol, captopril, ramipril, sildenafil, vitamin D, and 17β-estradiol. Among these, ACE inhibitors are established antihypertensive agents, while metformin exhibits protective effects in the context of diabetes and obesity [239,240]. Although these drugs have demonstrated benefits against specific CKM-related conditions in adult offspring [241,242], it remains unclear whether these effects are mediated via H_2_S-dependent mechanisms.

**Figure 3:**
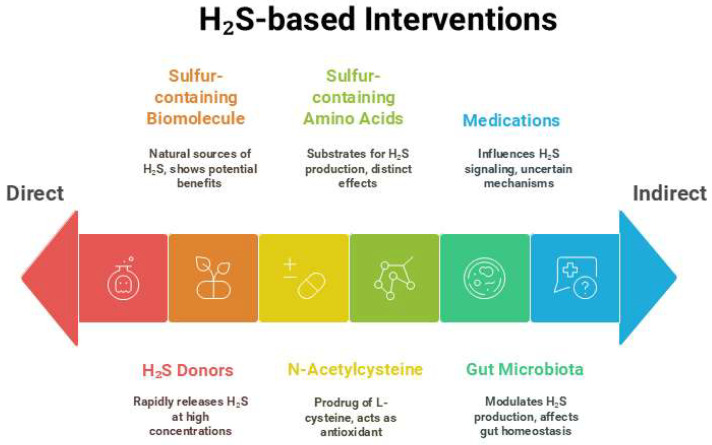
Schematic illustration of H_2_S-based interventions, from direct to indirect effects, aimed at preventing cardiovascular–kidney–metabolic syndrome.

Several important questions remain to be addressed, such as the therapeutic versus toxic concentrations of H_2_S and its metabolic derivatives, and the mechanisms by which these drugs influence or release H_2_S. Clarifying these gaps is essential to harness the H_2_S-modulating potential of existing pharmacotherapies in order to interrupt disease programming pathways and develop preventive strategies for CKM syndrome. H_2_S-based interventions aimed at preventing CKM syndrome, ranging from direct to indirect effects, are illustrated in Figure 3.

## 7. Conclusions and Perspectives

**Figure 4:**
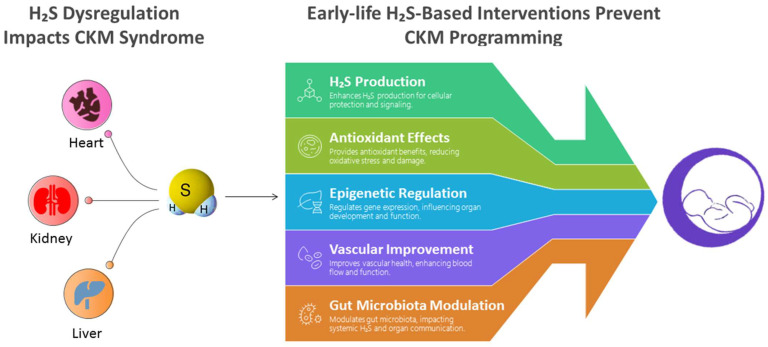
Schematic illustration of how H_2_S dysregulation contributes to cardiovascular–kidney–metabolic (CKM) syndrome, and how early-life H_2_S-based interventions can prevent CKM programming through protective mechanisms.

H_2_S appears to play a critical role in health and disease across the lifespan. In addition to its well-established involvement in adult CKM syndrome, the current literature reviewed here highlights that dysregulated H_2_S signaling is also evident in early life, contributing to the developmental programming of CKM conditions. The importance of H_2_S-based interventions during gestation and lactation is underscored by evidence from various animal models, in which sulfur-containing amino acids, NAC, H_2_S donors, and other sulfur-containing biomolecules have shown protective effects against CKM-related outcomes in offspring. Figure 4 summarizes how H_2_S dysregulation contributes to CKM syndrome and how early-life H_2_S treatment can prevent CKM programming.

Despite these advances, most studies to date have primarily focused on direct H_2_S-based interventions, while the role of gut microbiota-derived H_2_S in CKM programming remains poorly understood. It is still unclear whether gut-derived H_2_S exerts beneficial or harmful effects on CKM health, and whether microbiota-targeted strategies—such as modulation of sulfate-reducing and sulfur-oxidizing bacteria—could influence systemic H_2_S availability and impact developmental programming. This represents an important area for future investigation.

Another relatively unexplored dimension of H_2_S biology is its interaction with epigenetic regulatory mechanisms [43], particularly during fetal development. H_2_S has the potential to regulate gene expression through epigenetic modifications across multiple organs, potentially interacting with other molecular pathways to either promote or reverse CKM programming. A critical gap in this field is the limited exploration of persulfidation proteomics [243]. Although numerous studies have demonstrated protein S-persulfidation, its role in human diseases—including CKM syndrome—remains largely unclear. Persulfidation proteomics represents a promising strategy to delineate how H_2_S-mediated post-translational modifications regulate cellular processes relevant to cardiovascular, kidney, and metabolic health [244]. Future efforts should prioritize the refinement of high-resolution, quantitative proteomic technologies to map dynamic persulfidation networks under physiological and pathological conditions. Integrating these profiles with other epigenetic and metabolic signatures may reveal novel regulatory circuits linking redox signaling to gene expression. Furthermore, identifying disease-specific persulfidation patterns could provide early diagnostic biomarkers and mechanistic insights into disorders such as diabetes, CVD, and CKD. Ultimately, advances in this area may pave the way for targeted H_2_S-based interventions and precision medicine strategies.

It is noteworthy that the interplay between H_2_S and other gasotransmitters, such as NO and CO, is critical for human health and disease [245,246,247]. While we previously discussed this interplay in relation to kidney programming [248], we chose not to examine it further in the current review. Therefore, readers are encouraged to consult other reviews for more detailed information if they wish.

To enhance the real-world impact of early-life interventions, future studies should be strategically embedded within established maternal and child health systems, as well as national nutritional policy frameworks. Incorporating H_2_S-based interventions into routine prenatal care and early childhood dietary guidelines holds promise as an accessible and cost-efficient strategy for mitigating CKM syndrome risk [249,250]. Broad adoption may be further supported by embedding these interventions into local public health infrastructures, maternal health education programs, and government-led health promotion efforts—particularly in communities facing heightened vulnerability or limited access to care [251,252].

It is also important to recognize that while physiological levels of H_2_S are beneficial, supraphysiological concentrations can be toxic. Translational research, including well-designed clinical trials, is needed to determine whether the promising findings from preclinical models can be effectively applied to human populations. Efforts should focus not only on enhancing the therapeutic efficacy of H_2_S-based interventions for treating CKM syndrome but, more importantly, on developing strategies for early-life prevention.

## Figures and Tables

**Table 1:** Summary of H_2_S-based interventions utilized as reprogramming strategies in animal models for preventing offspring CKM syndrome.

H_2_S-Based Intervention	Supplementation Period	Animal Models	Species/Gender	Age at Evaluation (Weeks)	Prevented CKM Conditions	Ref.
Sulfur-containing amino acids						
L-cysteine (8 mmol/kg/day)	Pregnancy	Maternal CKD	SD rat/M	12	Hypertension and kidney disease	[198]
D-cysteine (8 mmol/kg/day)	Pregnancy	Maternal CKD	SD rat/M	12	Hypertension and kidney disease	[198]
Taurine (200 mg/kg/day) daily intragastric administration	Pregnancy	Gestational diabetes	SD rat/M+F	8	Liver steatosis	[199]
1.5% taurine in drinking water	Pregnancy + Lactation	Maternal cafeteria diet	Wistar rat/M+F	20	Obesity and dyslipidemia	[200]
3% taurine in drinking water	Pregnancy + Lactation	Maternal high-sugar diet	SD rat/F	8	Hypertension and kidney disease	[201]
3% taurine in drinking water	Pregnancy + Lactation	Genetic hypertension model	SHR/M	22	Hypertension	[163]
5% taurine in chow	Pregnancy + Lactation	Genetic hypertension model	SHRSP/M	12	Hypertension	[202]
*N*-acetylcysteine						
1% NAC in drinking water	Pregnancy + Lactation	Prenatal dexamethasone plus post-weaning high-fat diet	SD rat/M	12	Hypertension and kidney disease	[203]
1% NAC in drinking water	Pregnancy + Lactation	Maternal L-NAME Exposure	SD rat/M	12	Hypertension and kidney disease	[204]
1% NAC in drinking water	Pregnancy + Lactation	Maternal suramin administration	SD rat/M	12	Hypertension	[205]
1% NAC in drinking water	Pregnancy + Lactation	Maternal hypertension	SHR rat/M	12	Hypertension	[206]
NAC (500 mg/kg/day) in drinking water	Gestational day 4 to postnatal day 10	Maternal nicotine exposure	SD rat/M	32	Hypertension and kidney disease	[207]
NAC (400 mg/kg/day) in drinking water	Pregnancy + Lactation	Postnatal high-fat diet	C57Bl6/J mice/M+F	16	Glucose intolerance	[208]
NAC (300 mg/kg/day) in drinking water	Lactation	Maternal high-fat diet	ICR-CD1/M+F	16	Dyslipidemia and liver steatosis	[209]
H_2_S donors						
NaHS (56 μmol/kg/day) daily intraperitoneal injection	Pregnancy + Lactation	2-kidney, 1-clip renovascular hypertension model	SD rat/M and F	16	Hypertension	[210,211]
Sodium thiosulfate (2 g/kg/day) in drinking water	Pregnancy + Lactation	Maternal CKD	SD rat/M	12	Hypertension	[212]
Sulfur-containing biomolecules						
Garlic oil (100 mg/kg/day)	Pregnancy + Lactation	Maternal CKD	SD rat/M	12	Hypertension	[213]
Garlic oil (100 mg/kg/day)	Pregnancy + Lactation	Maternal high-fat diet	SD rat/M	16	Hypertension	[214]
3% chondroitin sulfate in chow	Pregnancy + Lactation	Maternal CKD	SD rat/M	12	Hypertension	[215]

NAC = *N*-acetylcysteine. NaHS = sodium hydrosulfide. CKD = chronic kidney disease. L-NAME = N^G^-nitro-L-arginine-methyl ester. SHR = spontaneously hypertensive rat. SD = Sprague–Dawley. SHRSP = stroke-prone spontaneously hypertensive rat. M = male. F = female.

## Data Availability

Data are contained within the article.

## References

[1] Reiffenstein R.J., Hulbert W.C., Roth S.H. (1992). Toxicology of hydrogen sulfide. Annu. Rev. Pharmacol. Toxicol..

[2] Malone Rubright S.L., Pearce L.L., Peterson J. (2017). Environmental toxicology of hydrogen sulfide. Nitric Oxide.

[3] Szabo C. (2018). A timeline of hydrogen sulfide (H_2_S) research: From environmental toxin to biological mediator. Biochem. Pharmacol..

[4] Wang R. (2010). Hydrogen sulfide: The third gasotransmitter in biology and medicine. Antioxid. Redox Signal..

[5] Olson K.R., Straub K.D. (2016). The role of hydrogen sulfide in evolution and the evolution of hydrogen sulfide in metabolism and signaling. Physiology.

[6] Hughes M.N., Centelles M.N., Moore K.P. (2009). Making and working with hydrogen sulfide: The chemistry and generation of hydrogen sulfide in vitro and its measurement in vivo: A review. Free Radic. Biol. Med..

[7] Nagy P., Pálinkás Z., Nagy A., Budai B., Tóth I., Vasas A. (2014). Chemical aspects of hydrogen sulfide measurements in physiological samples. Biochim. Biophys. Acta.

[8] Dawson P.A., Elliott A., Bowling F.G. (2015). Sulphate in pregnancy. Nutrients.

[9] Chen C.J., Cheng M.C., Hsu C.N., Tain Y.L. (2023). Sulfur-Containing Amino Acids, Hydrogen Sulfide, and Sulfur Compounds on Kidney Health and Disease. Metabolites.

[10] Hanson M. (2015). The birth and future health of DOHaD. J. Dev. Orig. Health Dis..

[11] Jaradat J.H., Nashwan A.J. (2023). Cardiovascular-kidney-metabolic syndrome: Understanding the interconnections and the need for holistic intervention. J. Med. Surg. Public Health.

[12] Ndumele C.E., Rangaswami J., Chow S.L., Neeland I.J., Tuttle K.R., Khan S.S., Coresh J., Mathew R.O., Baker-Smith C.M., Carnethon M.R. (2023). Cardiovascular-Kidney-Metabolic Health: A Presidential Advisory from the American Heart Association. Circulation.

[13] Aggarwal R., Ostrominski J.W., Vaduganathan M. (2024). Prevalence of Cardiovascular-Kidney-Metabolic Syndrome Stages in US Adults, 2011–2020. JAMA.

[14] Tain Y.L., Lin Y.J., Hsu C.N. (2025). Animal Models for Studying Developmental Origins of Cardiovascular-Kidney-Metabolic Syndrome. Biomedicines.

[15] Paauw N.D., van Rijn B.B., Lely A.T., Joles J.A. (2017). Pregnancy as a critical window for blood pressure regulation in mother and child: Programming and reprogramming. Acta Physiol..

[16] Desai K.M., Chang T., Untereiner A., Wu L. (2011). Hydrogen sulfide and the metabolic syndrome. Expert Rev. Clin. Pharmacol..

[17] Bełtowski J., Jamroz-Wiśniewska A. (2016). Hydrogen Sulfide in the Adipose Tissue-Physiology, Pathology and a Target for Pharmacotherapy. Molecules.

[18] Peleli M., Zampas P., Papapetropoulos A. (2022). Hydrogen Sulfide and the Kidney: Physiological Roles, Contribution to Pathophysiology, and Therapeutic Potential. Antioxid. Redox Signal..

[19] Kolluru G.K., Shackelford R.E., Shen X., Dominic P., Kevil C.G. (2023). Sulfide regulation of cardiovascular function in health and disease. Nat. Rev. Cardiol..

[20] Kimura H. (2021). Hydrogen Sulfide (H_2_S) and Polysulfide (H_2_Sn) Signaling: The First 25 Years. Biomolecules.

[21] Warenycia M.W., Goodwin L.R., Benishin C.G., Reiffenstein R.J., Grancom D.M., Taylor J.D., Dieken F.P. (1989). Acute hydrogen sulfide poisoning. Demonstration of selective uptake of sulfide by the brainstem by measurement of brain sulfide levels. Biochem. Pharmacol..

[22] Kajimura M., Fukuda R., Bateman R.M., Yamamoto T., Suematsu M. (2010). Interactions of multiple gas-transducing systems: Hallmarks and uncertainties of CO, NO, and H_2_S gas biology. Antioxid. Redox Signal..

[23] Cirino G., Szabo C., Papapetropoulos A. (2023). Physiological roles of hydrogen sulfide in mammalian cells, tissues, and organs. Physiol. Rev..

[24] Shibuya N., Koike S., Tanaka M., Ishigami-Yuasa M., Kimura Y., Ogasawara Y., Fukui K., Nagahara N., Kimura H. (2013). A novel pathway for the production of hydrogen sulfide from D-cysteine in mammalian cells. Nat. Commun..

[25] Schmitz R.A., Mohammadi S.S., van Erven T., Berben T., Jetten M.S.M., Pol A., Op den Camp H.J.M. (2022). Methanethiol Consumption and Hydrogen Sulfide Production by the Thermoacidophilic Methanotroph Methylacidiphilum fumariolicum SolV. Front. Microbiol..

[26] Giuffrè A., Tomé C.S., Fernandes D.G.F., Zuhra K., Vicente J.B. (2020). Hydrogen Sulfide Metabolism and Signaling in the Tumor Microenvironment. Adv. Exp. Med. Biol..

[27] Yang G., Wu L. (2017). Trend in H_2_S Biology and Medicine Research—A Bibliometric Analysis. Molecules.

[28] Benavides G.A., Squadrito G.L., Mills R.W., Patel H.D., Isbell T.S., Patel R.P., Darley-Usmar V.M., Doeller J.E., Kraus D.W. (2007). Hydrogen sulfide mediates the vasoactivity of garlic. Proc. Natl. Acad. Sci. USA.

[29] Fukuto J.M., Ignarro L.J., Nagy P., Wink D.A., Kevil C.G., Feelisch M., Cortese-Krott M.M., Bianco C.L., Kumagai Y., Hobbs A.J. (2018). Biological hydropersulfides and related polysulfides—A new concept and perspective in redox biology. FEBS Lett..

[30] Khodade V.S., Aggarwal S.C., Eremiev A., Bao E., Porche S., Toscano J.P. (2022). Development of Hydropersulfide Donors to Study Their Chemical Biology. Antioxid. Redox Signal..

[31] Zarenkiewicz J., Perez-Ternero C., Kojasoy V., McGinity C., Khodade V.S., Lin J., Tantillo D.J., Toscano J.P., Hobbs A.J., Fukuto J.M. (2022). The reaction of hydropersulfides (RSSH) with S-nitrosothiols (RS-NO) and the biological/physiological implications. Free Radic. Biol. Med..

[32] Pharoah B.M., Khodade V.S., Eremiev A., Bao E., Liu T., O’Rourke B., Paolocci N., Toscano J.P. (2022). Hydropersulfides (RSSH) Outperform Post-Conditioning and Other Reactive Sulfur Species in Limiting Ischemia-Reperfusion Injury in the Isolated Mouse Heart. Antioxidants.

[33] Pharoah B.M., Zhang C., Khodade V.S., Keceli G., McGinity C., Paolocci N., Toscano J.P. (2023). Hydropersulfides (RSSH) attenuate doxorubicin-induced cardiotoxicity while boosting its anticancer action. Redox Biol..

[34] Blachier F., Davila A.-M., Mimoun S., Benetti P.-H., Atanasiu C., Andriamihaja M., Benamouzig R., Bouillaud F., Tomé D. (2009). Luminal sulfide and large intestine mucosa: Friend or foe?. Amino Acids.

[35] Filipovic M.R., Zivanovic J., Alvarez B., Banerjee R. (2018). Chemical biology of H_2_S signaling through persulfidation. Chem. Rev..

[36] Meng G., Zhao S., Xie L., Han Y., Ji Y. (2018). Protein S-sulfhydration by hydrogen sulfide in cardiovascular system. Br. J. Pharmacol..

[37] Liu X.Y., Qian L.L., Wang R.X. (2022). Hydrogen Sulfide-Induced Vasodilation: The Involvement of Vascular Potassium Channels. Front. Pharmacol..

[38] Borisov V.B., Forte E. (2021). Impact of Hydrogen Sulfide on Mitochondrial and Bacterial Bioenergetics. Int. J. Mol. Sci..

[39] Lian J., Chen Y., Zhang Y., Guo S., Wang H. (2024). The role of hydrogen sulfide regulation of ferroptosis in different diseases. Apoptosis.

[40] Zhang M., Tong Z., Wang Y., Fu W., Meng Y., Huang J., Sun L. (2023). Relationship between ferroptosis and mitophagy in renal fibrosis: A systematic review. J. Drug Target..

[41] Wu Z.F., Yan B.J., Luo W., Gui D.D., Ren Z., Ma Y., Jiang Z.S. (2023). Ferroptosis and Hydrogen Sulfide in Cardiovascular Disease. Curr. Med. Chem..

[42] Sha W., Hu F., Xi Y., Chu Y., Bu S. (2021). Mechanism of Ferroptosis and Its Role in Type 2 Diabetes Mellitus. J. Diabetes Res..

[43] Spezzini J., Piragine E., d’Emmanuele di Villa Bianca R., Bucci M., Martelli A., Calderone V. (2023). Hydrogen sulfide and epigenetics: Novel insights into the cardiovascular effects of this gasotransmitter. Br. J. Pharmacol..

[44] Prasher D., Greenway S.C., Singh R.B. (2020). The impact of epigenetics on cardiovascular disease. Biochem. Cell Biol..

[45] Leucker T.M., Nomura Y., Kim J.H., Bhatta A., Wang V., Wecker A., Jandu S., Santhanam L., Berkowitz D., Romer L. (2017). Cystathionine γ-lyase protects vascular endothelium: A role for inhibition of histone deacetylase 6. Am. J. Physiol. Heart Circ. Physiol..

[46] Lu Q.B., Ding Y., Fu X., Sun H.J., Zhang J.R. (2023). Hydrogen sulfide in health and diseases: Cross talk with noncoding RNAs. Am. J. Physiol. Cell Physiol..

[47] Liu J., Hao D.D., Zhang J.S., Zhu Y.C. (2011). Hydrogen sulphide inhibits cardiomyocyte hypertrophy by up-regulating miR-133a. Biochem. Biophys. Res. Commun..

[48] Yang G., Pei Y., Cao Q., Wang R. (2012). MicroRNA-21 represses human cystathionine gamma-lyase expression by targeting at specificity protein-1 in smooth muscle cells. J. Cell Physiol..

[49] Pushpakumar S., Kundu S., Weber G., Sen U. (2021). Exogenous hydrogen sulfide and miR-21 antagonism attenuates macrophage-mediated inflammation in ischemia reperfusion injury of the aged kidney. Geroscience.

[50] Dilek N., Papapetropoulos A., Toliver-Kinsky T., Szabo C. (2020). Hydrogen sulfide: An endogenous regulator of the immune system. Pharmacol. Res..

[51] Rose P., Moore P.K., Whiteman M., Kirk C., Zhu Y.Z. (2021). Diet and Hydrogen Sulfide Production in Mammals. Antioxid. Redox Signal..

[52] Marshall N.E., Abrams B., Barbour L.A., Catalano P., Christian P., Friedman J.E., Hay W.W., Hernandez T.L., Krebs N.F., Oken E. (2022). The importance of nutrition in pregnancy and lactation: Lifelong consequences. Am. J. Obstet. Gynecol..

[53] Pilsova A., Pilsova Z., Klusackova B., Zelenkova N., Chmelikova E., Postlerova P., Sedmikova M. (2024). Hydrogen sulfide and its role in female reproduction. Front. Vet. Sci..

[54] Tain Y.L., Hsu C.N. (2023). The Impact of Nutrient Intake and Metabolic Wastes during Pregnancy on Offspring Hypertension: Challenges and Future Opportunities. Metabolites.

[55] Brand E. (1946). Amino acid composition of simple proteins. Ann. N. Y. Acad. Sci..

[56] Kalhan S.C. (2016). One carbon metabolism in pregnancy: Impact on maternal, fetal and neonatal health. Mol. Cell. Endocrinol..

[57] Rees W.D., Hay S.M., Cruickshank M. (2006). An imbalance in the methionine content of the maternal diet reduces postnatal growth in the rat. Metabolism.

[58] Dasarathy J., Gruca L.L., Bennett C., Parimi P.S., Duenas C., Marczewski S., Fierro J.L., Kalhan S.C. (2010). Methionine metabolismin human pregnancy. Am. J. Clin. Nutr..

[59] Guerra D.D., Hurt K.J. (2019). Gasotransmitters in pregnancy: From conception to uterine involution. Biol. Reprod..

[60] Knapen M.F., Zusterzeel P.L., Peters W.H., Steegers E.A. (1999). Glutathione and glutathione-related enzymes in reproduction. A review. Eur. J. Obstet. Gynecol. Reprod. Biol..

[61] Viskova H., Vesela K., Janosikova B., Krijt J., Visek J.A., Calda P. (2007). Plasma cysteine concentrations in uncomplicated pregnancies. Fetal Diagn. Ther..

[62] Gaiday A.N., Tussupkaliyev A.B., Bermagambetova S.K., Zhumagulova S.S., Sarsembayeva L.K., Dossimbetova M.B., Daribay Z.Z. (2018). Effect of homocysteine on pregnancy: A systematic review. Chem. Biol. Interact..

[63] Tochitani S. (2022). Taurine: A Maternally Derived Nutrient Linking Mother and Offspring. Metabolites.

[64] Lerdweeraphon W., Wyss J.M., Boonmars T., Roysommuti S. (2013). Perinatal taurine exposure affects adult oxidative stress. Am. J. Physiol. Regul. Integr. Comp. Physiol..

[65] Linden D.R. (2014). Hydrogen Sulfide Signaling in the Gastrointestinal Tract. Antioxid. Redox Signal..

[66] Kimura H. (2015). Signaling molecules: Hydrogen sulfide and polysulfide. Antioxid. Redox Signal..

[67] Dawson P.A. (2011). Sulfate in fetal development. Semin. Cell Dev. Biol..

[68] Strott C.A. (2002). Sulfonation and molecular action. Endocr. Rev..

[69] Dawson P.A., Rakoczy J., Simmons D.G. (2012). Placental, renal, and ileal sulfate transporter gene expression in mouse gestation. Biol. Reprod..

[70] Lu Y., Zhang M., Huang D. (2022). Dietary Organosulfur-Containing Compounds and Their Health-Promotion Mechanisms. Annu. Rev. Food Sci. Technol..

[71] Barba F.J., Orlien V. (2017). Processing, bioaccessibility and bioavailability of bioactive sulfur compounds: Facts and gaps. J. Food Compos. Anal..

[72] Myhre R., Brantsæter A.L., Myking S., Eggesbø M., Meltzer H.M., Haugen M., Jacobsson B. (2013). Intakes of garlic and dried fruits are associated with lower risk of spontaneous preterm delivery. J. Nutr..

[73] Shang A., Cao S.Y., Xu X.Y., Gan R.Y., Tang G.Y., Corke H., Mavumengwana V., Li H.B. (2019). Bioactive Compounds and Biological Functions of Garlic (*Allium sativum* L.). Foods.

[74] You X.J., Xu C., Lu J.Q., Zhu X.Y., Gao L., Cui X.R., Li Y., Gu H., Ni X. (2011). Expression of cystathionine β-synthase and cystathionine γ-lyase in human pregnant myometrium and their roles in the control of uterine contractility. PLoS ONE.

[75] Patel P., Vatish M., Heptinstall J., Wang R., Carson R. (2009). The endogenous production of hydrogen sulphide in intrauterine tissues. Reprod. Biol. Endocrinol..

[76] Wang X., Tang J. (2023). Focal adhesion kinase signaling is necessary for the hydrogen sulfide-enhanced proliferation, migration, and invasion of HTR8/SVneo human trophoblasts. Reprod. Dev. Med..

[77] Hu R., Lu J., You X., Zhu X., Hui N., Ni X. (2011). Hydrogen sulfide inhibits the spontaneous and oxytocin-induced contractility of human pregnant myometrium. Gynecol. Endocrinol..

[78] Holwerda K.M., Bos E.M., Rajakumar A., Ris-Stalpers C., van Pampus M.G., Timmer A., Erwich J.J., Faas M.M., van Goor H., Lely A.T. (2012). Hydrogen sulfide producing enzymes in pregnancy and preeclampsia. Placenta.

[79] d’Emmanuele di Villa Bianca R., Fusco F., Mirone V., Cirino G., Sorrentino R. (2017). The Role of the Hydrogen Sulfide Pathway in Male and Female Urogenital System in Health and Disease. Antioxid. Redox Signal..

[80] Aubard Y., Darodes N., Cantaloube M. (2000). Hyperhomocysteinemia and pregnancy—Review of our present understanding and therapeutic implications. Eur. J. Obstet. Gynecol. Reprod. Biol..

[81] Carson R., Konje J. (2014). Role of hydrogen sulfide in the female reproductive tract. Expert Rev. Obstet. Gynecol..

[82] Lin X., Li H. (2021). Obesity: Epidemiology, Pathophysiology, and Therapeutics. Front. Endocrinol..

[83] Hagberg C.E., Spalding K.L. (2024). White adipocyte dysfunction and obesity-associated pathologies in humans. Nat. Rev. Mol. Cell Biol..

[84] Flori L., Piragine E., Calderone V., Testai L. (2024). Role of hydrogen sulfide in the regulation of lipid metabolism: Implications on cardiovascular health. Life Sci..

[85] Zhang H., Huang Y., Chen S., Tang C., Wang G., Du J., Jin H. (2020). Hydrogen sulfide regulates insulin secretion and insulin resistance in diabetes mellitus, a new promising target for diabetes mellitus treatment? A review. J. Adv. Res..

[86] Zhu L., Yang B., Ma D., Wang L., Duan W. (2020). Hydrogen Sulfide, Adipose Tissue and Diabetes Mellitus. Diabetes Metab. Syndr. Obes..

[87] Mateus I., Prip-Buus C. (2022). Hydrogen sulphide in liver glucose/lipid metabolism and non-alcoholic fatty liver disease. Eur. J. Clin. Investig..

[88] Woo C.W., Siow Y.L., Pierce G.N., Choy P.C., Minuk G.Y., Mymin D., O K. (2005). Hyperhomocysteinemia induces hepatic cholesterol biosynthesis and lipid accumulation via activation of transcription factors. Am. J. Physiol. Endocrinol. Metab..

[89] Werstuck G.H., Lentz S.R., Dayal S., Hossain G.S., Sood S.K., Shi Y.Y., Zhou J., Maeda N., Krisans S.K., Malinow M.R. (2001). Homocysteine-induced endoplasmic reticulum stress causes dysregulation of the cholesterol and triglyceride biosynthetic pathways. J. Clin. Investig..

[90] Luo Z.L., Tang L.J., Wang T., Dai R.W., Ren J.D., Cheng L., Xiang K., Tian F.Z. (2014). Effects of treatment with hydrogen sulfide on methionine-choline deficient diet-induced non-alcoholic steatohepatitis in rats. J. Gastroenterol. Hepatol..

[91] Peh M.T., Anwar A.B., Ng D.S.W., Atan M.S.B.M., Kumar S.D., Moore P.K. (2014). Effect of feeding a high fat diet on hydrogen sulfide (H_2_S) metabolism in the mouse. Nitric Oxide.

[92] Wu D., Zheng N., Qi K., Cheng H., Sun Z., Gao B., Zhang Y., Pang W., Huangfu C., Ji S. (2015). Exogenous hydrogen sulfide mitigates the fatty liver in obese mice through improving lipid metabolism and antioxidant potential. Med. Gas Res..

[93] Zhang N., Wang Y., Zhang J., Liu B., Li G., Xin S., Xu K. (2019). Diallyl disulfide attenuates non-alcoholic steatohepatitis by suppressing key regulators of lipid metabolism, lipid peroxidation and inflammation in mice. Mol. Med. Rep..

[94] Xia M., Chen L., Muh R.W., Li P.L., Li N. (2009). Production and actions of hydrogen sulfide, a novel gaseous bioactive substance, in the kidneys. J. Pharmacol. Exp. Ther..

[95] Ge S.N., Zhao M.M., Wu D.D., Chen Y., Wang Y., Zhu J.H., Cai W.J., Zhu Y.Z., Zhu Y.C. (2014). Hydrogen sulfide targets EGFR Cys797/Cys798 residues to induce Na^+^/K^+^-ATPase endocytosis and inhibition in renal tubular epithelial cells and increase sodium excretion in chronic salt-loaded rats. Antioxid. Redox Signal..

[96] Kuang Q., Xue N., Chen J., Shen Z., Cui X., Fang Y., Ding X. (2018). Low plasma hydrogen sulfide is associated with impaired renal function and cardiac dysfunction. Am. J. Nephrol..

[97] Lu M., Liu Y.H., Goh H.S., Wang J.J., Yong Q.C., Wang R., Bian J.S. (2010). Hydrogen sulfide inhibits plasma renin activity. J. Am. Soc. Nephrol..

[98] Van Goor H., van den Born J.C., Hillebrands J.L., Joles J.A. (2016). Hydrogen sulfide in hypertension. Curr. Opin. Nephrol. Hypertens..

[99] Lv B., Chen S., Tang C., Jin H., Du J., Huang Y. (2020). Hydrogen sulfide and vascular regulation—An update. J. Adv. Res..

[100] Lo Faro M.L., Fox B., Whatmore J.L., Winyard P.G., Whiteman M. (2014). Hydrogen sulfide and nitric oxide interactions in inflammation. Nitric Oxide.

[101] Chen T., Tian M., Han Y. (2020). Hydrogen sulfide: A multi-tasking signal molecule in the regulation of oxidative stress responses. J. Exp. Bot..

[102] Dugbartey G.J. (2023). Physiological role of hydrogen sulfide in the kidney and its therapeutic implications for kidney diseases. Biomed. Pharmacother..

[103] Szlęzak D., Hutsch T., Ufnal M., Wróbel M. (2022). Heart and kidney H_2_S production is reduced in hypertensive and older rats. Biochimie.

[104] Bełtowski J., Kowalczyk-Bołtuć J. (2023). Hydrogen sulfide in the experimental models of arterial hypertension. Biochem. Pharmacol..

[105] Citi V., Martelli A., Bucci M., Piragine E., Testai L., Vellecco V., Cirino G., Calderone V. (2020). Searching for novel hydrogen sulfide donors: The vascular effects of two thiourea derivatives. Pharmacol. Res..

[106] Xiao L., Dong J.H., Jin S., Xue H.M., Guo Q., Teng X., Wu Y.M. (2016). Hydrogen sulfide improves endothelial dysfunction via downregulating BMP4/COX-2 pathway in rats with hypertension. Oxid. Med. Cell. Longev..

[107] Zhong G., Chen F., Cheng Y., Tang C., Du J. (2003). The role of hydrogen sulfide generation in the pathogenesis of hypertension in rats induced by inhibition of nitric oxide synthase. J. Hypertens..

[108] Huang P., Chen S., Wang Y., Liu J., Yao Q., Huang Y., Li H., Zhu M., Wang S., Li L. (2015). Down-regulated CBS/H_2_S pathway is involved in high-salt-induced hypertension in Dahl rats. Nitric Oxide.

[109] Yang G., Wu L., Jiang B., Yang W., Qi J., Cao K., Meng Q., Mustafa A.K., Mu W., Zhang S. (2008). H_2_S as a physiologic vasorelaxant: Hypertension in mice with deletion of cystathionine gamma-lyase. Science.

[110] Ishii I., Akahoshi N., Yamada H., Nakano S., Izumi T., Suematsu M. (2010). Cystathionine gamma-Lyase-deficient mice require dietary cysteine to protect against acute lethal myopathy and oxidative injury. J. Biol. Chem..

[111] Perna A.F., Ingrosso D. (2012). Low hydrogen sulphide and chronic kidney disease: A dangerous liaison. Nephrol. Dial. Transp..

[112] Cao X., Bian J.S. (2016). The Role of Hydrogen Sulfide in Renal System. Front. Pharmacol..

[113] Dugbartey G.J. (2017). H_2_S as a possible therapeutic alternative for the treatment of hypertensive kidney injury. Nitric Oxide.

[114] Citi V., Martelli A., Gorica E., Brogi S., Testai L., Calderone V. (2021). Role of hydrogen sulfide in endothelial dysfunction: Pathophysiology and therapeutic approaches. J. Adv. Res..

[115] Gemici B., Elsheikh W., Feitosa K.B., Costa S.K., Muscara M.N., Wallace J.L. (2015). H_2_S-releasing drugs: Anti-inflammatory, cytoprotective and chemopreventative potential. Nitric Oxide.

[116] Mani S., Untereiner A., Wu L., Wang R. (2014). Hydrogen sulfide and the pathogenesis of atherosclerosis. Antioxid. Redox Signal..

[117] Gorini F., Bustaffa E., Chatzianagnostou K., Bianchi F., Vassalle C. (2020). Hydrogen sulfide and cardiovascular disease: Doubts, clues, and interpretation difficulties from studies in geothermal areas. Sci. Total Environ..

[118] Li Z., Polhemus D.J., Lefer D.J. (2018). Evolution of hydrogen sulfide therapeutics to treat cardiovascular disease. Circ. Res..

[119] Zhang L., Wang Y., Li Y., Li L., Xu S., Feng X., Liu S. (2018). Hydrogen sulfide (H_2_S)-releasing compounds: Therapeutic potential in cardiovascular diseases. Front. Pharmacol..

[120] Rajendran S., Shen X., Glawe J., Kolluru G.K., Kevil C.G. (2019). Nitric oxide and hydrogen sulfide regulation of ischemic vascular growth and remodeling. Compr. Physiol..

[121] Ding Q., Song W., Zhu M., Yu Y., Lin Z., Hu W., Cai J., Zhang Z., Zhang H., Zhou J. (2024). Hydrogen Sulfide and Functional Therapy: Novel Mechanisms from Epigenetics. Antioxid. Redox Signal..

[122] Hsu C.N., Tain Y.L. (2021). Preventing Developmental Origins of Cardiovascular Disease: Hydrogen Sulfide as a Potential Target?. Antioxidants.

[123] Huang D., Jing G., Zhu S. (2023). Regulation of Mitochondrial Respiration by Hydrogen Sulfide. Antioxidants.

[124] Paul B.D., Snyder S.H., Kashfi K. (2021). Effects of hydrogen sulfide on mitochondrial function and cellular bioenergetics. Redox Biol..

[125] Luo Y., Melhem S., Feelisch M., Chatre L., Morton N.M., Dolga A.M., van Goor H. (2025). Thiosulphate sulfurtransferase: Biological roles and therapeutic potential. Redox Biol..

[126] Landry A.P., Ballou D.P., Banerjee R. (2017). H_2_S oxidation by nanodisc-embedded human sulfide quinone oxidoreductase. J. Biol. Chem..

[127] Tiranti V., Viscomi C., Hildebrandt T., Di Meo I., Mineri R., Tiveron C., Levitt M.D., Prelle A., Fagiolari G., Rimoldi M. (2009). Loss of ETHE1, a mitochondrial dioxygenase, causes fatal sulfide toxicity in ethylmalonic encephalopathy. Nat. Med..

[128] Kleiner G., Barca E., Ziosi M., Emmanuele V., Xu Y., Hidalgo-Gutierrez A., Qiao C., Tadesse S., Area-Gomez E., Lopez L.C. (2018). CoQ10 supplementation rescues nephrotic syndrome through normalization of H_2_S oxidation pathway. Biochim. Biophys. Acta Mol. Basis Dis..

[129] Combi Z., Potor L., Nagy P., Sikura K.É., Ditrói T., Jurányi E.P., Galambos K., Szerafin T., Gergely P., Whiteman M. (2023). Hydrogen sulfide as an anti-calcification stratagem in human aortic valve: Altered biogenesis and mitochondrial metabolism of H_2_S lead to H_2_S deficiency in calcific aortic valve disease. Redox Biol..

[130] Ziosi M., Di Meo I., Kleiner G., Gao X.H., Barca E., Sanchez-Quintero M.J., Tadesse S., Jiang H., Qiao C., Rodenburg R.J. (2017). Coenzyme Q deficiency causes impairment of the sulfide oxidation pathway. EMBO Mol. Med..

[131] Bełtowski J. (2013). Endogenous hydrogen sulfide in perivascular adipose tissue: Role in the regulation of vascular tone in physiology and pathology. Can. J. Physiol. Pharmacol..

[132] Kruithof P.D., Lunev S., Aguilar Lozano S.P., de Assis Batista F., Al-Dahmani Z.M., Joles J.A., Dolga A.M., Groves M.R., van Goor H. (2020). Unraveling the role of thiosulfate sulfurtransferase in metabolic diseases. Biochim. Biophys. Acta Mol. Basis Dis..

[133] Hsu C.N., Tain Y.L. (2021). Animal Models for DOHaD Research: Focus on Hypertension of Developmental Origins. Biomedicines.

[134] Arima Y., Nishiyama K., Izumiya Y., Kaikita K., Hokimoto S., Tsujita K. (2018). Fetal Origins of Hypertension. Adv. Exp. Med. Biol..

[135] Nüsken E., Dötsch J., Weber L.T., Nüsken K.D. (2018). Developmental Programming of Renal Function and Re-Programming Approaches. Front. Pediatr..

[136] Kett M.M., Denton K.M. (2011). Renal programming: Cause for concern?. Am. J. Physiol. Regul. Integr. Comp. Physiol..

[137] McMillen I.C., Robinson J.S. (2005). Developmental origins of the metabolic syndrome: Prediction, plasticity, and programming. Physiol. Rev..

[138] Hoffman D.J., Powell T.L., Barrett E.S., Hardy D.B. (2021). Developmental origins of metabolic diseases. Physiol. Rev..

[139] Tain Y.L., Hsu C.N. (2017). Interplay between oxidative stress and nutrient sensing signaling in the developmental origins of cardiovascular disease. Int. J. Mol. Sci..

[140] Franco Mdo C., Ponzio B.F., Gomes G.N., Gil F.Z., Tostes R., Carvalho M.H., Fortes Z.B. (2009). Micronutrient prenatal supplementation prevents the development of hypertension and vascular endothelial damage induced by intrauterine malnutrition. Life Sci..

[141] Mas-Parés B., Xargay-Torrent S., Carreras-Badosa G., Gómez-Vilarrubla A., Niubó-Pallàs M., Tibau J., Reixach J., Prats-Puig A., de Zegher F., Ibañez L. (2024). Gestational Caloric Restriction Alters Adipose Tissue Methylome and Offspring’s Metabolic Profile in a Swine Model. Int. J. Mol. Sci..

[142] Ozanne S.E., Smith G.D., Tikerpae J., Hales C.N. (1996). Altered regulation of hepatic glucose output in the male offspring of protein-malnourished rat dams. Am. J. Physiol..

[143] Alejandro E.U., Jo S., Akhaphong B., Llacer P.R., Gianchandani M., Gregg B., Parlee S.D., MacDougald O.A., Bernal-Mizrachi E. (2020). Maternal low-protein diet on the last week of pregnancy contributes to insulin resistance and β-cell dysfunction in the mouse offspring. Am. J. Physiol. Regul. Integr. Comp. Physiol..

[144] Conceição E.P., Franco J.G., Oliveira E., Resende A.C., Amaral T.A., Peixoto-Silva N., Passos M.C., Moura E.G., Lisboa P.C. (2013). Oxidative stress programming in a rat model of postnatal early overnutrition--role of insulin resistance. J. Nutr. Biochem..

[145] Souza L.L., Moura E.G., Lisboa P.C. (2022). Litter Size Reduction as a Model of Overfeeding during Lactation and Its Consequences for the Development of Metabolic Diseases in the Offspring. Nutrients.

[146] Saad A.F., Dickerson J., Kechichian T.B., Yin H., Gamble P., Salazar A., Patrikeev I., Motamedi M., Saade G.R., Costantine M.M. (2016). High-fructose diet in pregnancy leads to fetal programming of hypertension, insulin resistance, and obesity in adult offspring. Am. J. Obstet. Gynecol..

[147] Seong H.Y., Cho H.M., Kim M., Kim I. (2019). Maternal High-Fructose Intake Induces Multigenerational Activation of the Renin-Angiotensin-Aldosterone System. Hypertension.

[148] Tsai T.A., Tsai C.K., HuAng L.T., Sheen J.M., Tiao M.M., Tain Y.L., Chen C.C., Lin I.C., Lai Y.J., Tsai C.C. (2020). Maternal Resveratrol Treatment Re-Programs and Maternal High-Fat Diet-Induced Retroperitoneal Adiposity in Male Offspring. Int. J. Environ. Res. Public Health.

[149] Peng H., Li J., Xu H., Wang X., He L., McCauley N., Zhang K.K., Xie L. (2023). Offspring NAFLD liver phospholipid profiles are differentially programmed by maternal high-fat diet and maternal one carbon supplement. J. Nutr. Biochem..

[150] Langley-Evans S.C. (2015). Nutrition in early life and the programming of adult disease: A review. J. Hum. Nutr. Diet..

[151] Parra-Vargas M., Bouret S.G., Bruning J.C., de Moura E.G., Garland T., Lisboa P.C., Ozanne S.E., Patti M.E., Plagemann A., Speakman J.R. (2023). The long-lasting shadow of litter size in rodents: Litter size is an underreported variable that strongly determines adult physiology. Mol. Metab..

[152] Johnson R.J., Segal M.S., Sautin Y., Nakagawa T., Feig D.I., Kang D.-H., Gersch M.S., Benner S., Sánchez-Lozada L.G. (2007). Potential role of sugar (fructose) in the epidemic of hypertension, obesity and the metabolic syndrome, diabetes, kidney disease, and cardiovascular disease. Am. J. Clin. Nutr..

[153] Williams L., Seki Y., Vuguin P.M., Charron M.J. (2014). Animal models of in utero exposure to a high fat diet: A review. Biochim. Biophys. Acta.

[154] Silver D.J., Roversi G.A., Bithi N., Wang S.Z., Troike K.M., Neumann C.K., Ahuja G.K., Reizes O., Brown J.M., Hine C. (2021). Severe consequences of a high-lipid diet include hydrogen sulfide dysfunction and enhanced aggression in glioblastoma. J. Clin. Investig..

[155] Roglans N., Fauste E., Bentanachs R., Velázquez A.M., Pérez-Armas M., Donis C., Panadero M.I., Alegret M., Otero P., Bocos C. (2022). Bempedoic Acid Restores Liver H_2_S Production in a Female Sprague-Dawley Rat Dietary Model of Non-Alcoholic Fatty Liver. Int. J. Mol. Sci..

[156] Fauste E., Rodrigo S., Aguirre R., Rodríguez L., Álvarez-Millán J.J., Panadero M.I., Otero P., Bocos C. (2021). Liquid carbohydrate intake modifies transsulfuration pathway both in pregnant rats and in their male descendants. Clin. Investig. Arterioscler..

[157] Moon J.H., Jang H.C. (2022). Gestational Diabetes Mellitus: Diagnostic Approaches and Maternal-Offspring Complications. Diabetes Metab. J..

[158] Srinivasan K., Ramarao P. (2007). Animal models in type 2 diabetes research: An overview. Indian J. Med. Res..

[159] Wu W., Tan Q.Y., Xi F.F., Ruan Y., Wang J., Luo Q., Dou X.B., Hu T.X. (2022). NLRP3 inflammasome activation in gestational diabetes mellitus placentas is associated with hydrogen sulfide synthetase deficiency. Exp. Ther. Med..

[160] Wichi R.B., Souza S.B., Casarini D.E., Morris M., Barreto-Chaves M.L., Irigoyen M.C. (2005). Increased blood pressure in the offspring of diabetic mothers. Am. J. Physiol. Regul. Integr. Comp. Physiol..

[161] Chen Y.W., Chenier I., Tran S., Scotcher M., Chang S.Y., Zhang S.L. (2010). Maternal diabetes programs hypertension and kidney injury in offspring. Pediatr. Nephrol..

[162] Tain Y.L., Lee W.C., Hsu C.N., Lee W.C., Huang L.T., Lee C.T., Lin C.Y. (2013). Asymmetric dimethylarginine is associated with developmental programming of adult kidney disease and hypertension in offspring of streptozotocin-treated mothers. PLoS ONE.

[163] Thaeomor A., TeAngphuck P., Chaisakul J., Seanthaweesuk S., Somparn N., Roysommuti S. (2017). Perinatal Taurine Supplementation Prevents Metabolic and Cardiovascular Effects of Maternal Diabetes in Adult Rat Offspring. Adv. Exp. Med. Biol..

[164] Wang G.L., Yuan H.J., Kong Q.Q., Zhang J., Han X., Gong S., Xu M.T., He N., Luo M.J., Tan J.H. (2024). High glucose exposure of preimplantation embryos causes glucose intolerance and insulin resistance in F1 and F2 male offspring. Biochim. Biophys. Acta. Mol. Basis Dis..

[165] Tain Y.L., Hou C.Y., ChAng-Chien G.P., Lin S., Hsu C.N. (2023). Protective Role of Taurine on Rat Offspring Hypertension in the Setting of Maternal Chronic Kidney Disease. Antioxidants.

[166] O’Dowd R., Kent J.C., Moseley J.M., Wlodek M.E. (2008). Effects of uteroplacental insufficiency and reducing litter size on maternal mammary function and postnatal offspring growth. Am. J. Physiol..

[167] Gonçalves G.D., Walton S.L., Gazzard S.E., van der Wolde J., Mathias P.C.F., Moritz K.M., Cullen-McEwen L.A., Bertram J.F. (2020). Maternal hypoxia developmentally programs low podocyte endowment in male, but not female offspring. Anat. Rec..

[168] Passos M.A., Passos M.C., Oliveira E., Trotta P.A., Nogueira-Neto J.F., Bonomo I.T., Lisboa P.C., de Moura E.G. (2011). Maternal prolactin inhibition during lactation is associated to renal dysfunction in their adult rat offspring. Horm. Metab. Res..

[169] Ingvorsen C., Brix S., Ozanne S.E., Hellgren L.I. (2015). The effect of maternal Inflammation on foetal programming of metabolic disease. Acta Physiol..

[170] Saif J., Ahmad S., Rezai H., Litvinova K., Sparatore A., Alzahrani F.A., Wang K., Ahmed A. (2021). Hydrogen sulfide releasing molecule MZe786 inhibits soluble Flt-1 and prevents preeclampsia in a refined RUPP mouse model. Redox Biol..

[171] Carlson Z., Hafner H., Mulcahy M., Bullock K., Zhu A., Bridges D., Bernal-Mizrachi E., Gregg B. (2020). Lactational metformin exposure programs offspring white adipose tissue glucose homeostasis and resilience to metabolic stress in a sex-dependent manner. Am. J. Physiol. Endocrinol. Metab..

[172] Schreuder M.F., Bueters R.R., Huigen M.C., Russel F.G., Masereeuw R., van den Heuvel L.P. (2011). Effect of drugs on renal development. Clin. J. Am. Soc. Nephrol..

[173] Slabiak-Blaz N., Adamczak M., Gut N., Grajoszek A., Nyengaard J.R., Ritz E., Wiecek A. (2015). Administration of cyclosporine a in pregnant rats—The effect on blood pressure and on the glomerular number in their offspring. Kidney Blood Press. Res..

[174] Tain Y.L., Li L.C., Kuo H.C., Chen C.J., Hsu C.N. (2025). Gestational Exposure to Nonsteroidal Anti-Inflammatory Drugs and Risk of Chronic Kidney Disease in Childhood. JAMA Pediatr..

[175] Celsi G., Kistner A., Aizman R., Eklöf A.C., Ceccatelli S., de Santiago A., Jacobson S.H. (1998). Prenatal dexamethasone causes oligonephronia, sodium retention, and higher blood pressure in the offspring. Pediatr. Res..

[176] O’Regan D., Kenyon C.J., Seckl J.R., Holmes M.C. (2004). Glucocorticoid exposure in late gestation in the rat permanently programs gender-specific differences in adult cardiovascular and metabolic physiology. Am. J. Physiol. Endocrinol. Metab..

[177] Kawakami-Mori F., Nishimoto M., Reheman L., Kawarazaki W., Ayuzawa N., Ueda K., Hirohama D., Kohno D., Oba S., Shimosawa T. (2018). Aberrant DNA methylation of hypothalamic angiotensin receptor in prenatal programmed hypertension. JCI Insight.

[178] Hong J.Y. (2022). Developmental Programming by Perinatal Glucocorticoids. Mol. Cells.

[179] d’Emmanuele di Villa Bianca R., Mitidieri E., Donnarumma E., Tramontano T., Brancaleone V., Cirino G., Bucci M., Sorrentino R. (2015). Hydrogen sulfide is involved in dexamethasone-induced hypertension in rat. Nitric Oxide.

[180] Li L., Whiteman M., Moore P.K. (2009). Dexamethasone inhibits lipopolysaccharide-induced hydrogen sulphide biosynthesis in intact cells and in an animal model of endotoxic shock. J. Cell. Mol. Med..

[181] Bełtowski J. (2015). Hydrogen sulfide in pharmacology and medicine—An update. Pharmacol. Rep..

[182] Zaorska E., Tomasova L., Koszelewski D., Ostaszewski R., Ufnal M. (2020). Hydrogen Sulfide in Pharmacotherapy, Beyond the Hydrogen Sulfide-Donors. Biomolecules.

[183] Puche-Juarez M., Toledano J.M., Moreno-Fernandez J., Gálvez-Ontiveros Y., Rivas A., Diaz-Castro J., Ochoa J.J. (2023). The Role of Endocrine Disrupting Chemicals in Gestation and Pregnancy Outcomes. Nutrients.

[184] LaKind J.S., Lehmann G.M., Davis M.H., Hines E.P., Marchitti S.A., Alcala C., Lorber M. (2018). Infant Dietary Exposures to Environmental Chemicals and Infant/Child Health: A Critical Assessment of the Literature. Environ. Health Perspect..

[185] Lite C., Raja G.L., Juliet M., Sridhar V.V., Subhashree K.D., Kumar P., Chakraborty P., Arockiaraj J. (2022). In utero exposure to endocrine-disrupting chemicals, maternal factors and alterations in the epigenetic landscape underlying later-life health effects. Environ. Toxicol. Pharmacol..

[186] Aragon A.C., Kopf P.G., Campen M.J., Huwe J.K., Walker M.K. (2008). In utero and lactational 2,3,7,8-tetrachlorodibenzo-p-dioxin exposure: Effects on fetal and adult cardiac gene expression and adult cardiac and renal morphology. Toxicol. Sci..

[187] Hsu C.N., Hung C.H., Hou C.Y., Chang C.I., Tain Y.L. (2021). Perinatal Resveratrol Therapy to Dioxin-Exposed Dams Prevents the Programming of Hypertension in Adult Rat Offspring. Antioxidants.

[188] Van Esterik J.C., Verharen H.W., Hodemaekers H.M., Gremmer E.R., Nagarajah B., Kamstra J.H., Dollé M.E., Legler J., vander Ven L.T. (2015). Compound- and sex-specific effects on programming of energy and immune homeostasis in adult C57BL/6JxFVB mice after perinatal TCDD and PCB 153. Toxicol. Appl. Pharmacol..

[189] Hsu C.N., Lin Y.J., Tain Y.L. (2019). Maternal exposure to bisphenol A combined with high-fat diet-induced programmed hypertension in adult male rat offspring: Effects of resveratrol. Int. J. Mol. Sci..

[190] Galyon K.D., Farshidi F., Han G., Ross M.G., Desai M., Jellyman J.K. (2017). Maternal bisphenol A exposure alters rat offspring hepatic and skeletal muscle insulin signaling protein abundance. Am. J. Obstet. Gynecol..

[191] Shih M.K., Tain Y.L., Chen Y.W., Hsu W.H., Yeh Y.T., Chang S.K.C., Liao J.X., Hou C.Y. (2021). Resveratrol Butyrate Esters Inhibit Obesity Caused by Perinatal Exposure to Bisphenol A in Female Offspring Rats. Molecules.

[192] Wei Z., Song L., Wei J., Chen T., Chen J., Lin Y., Xia W., Xu B., Li X., Chen X. (2012). Maternal exposure to di-(2-ethylhexyl) phthalate alters kidney development through the renin-Angiotensin system in offspring. Toxicol. Lett..

[193] Rajagopal G., Bhaskaran R.S., Karundevi B. (2019). Maternal di-(2-ethylhexyl) phthalate exposure alters hepatic insulin signal transduction and glucoregulatory events in rat F1 male offspring. J. Appl. Toxicol..

[194] Zhu Y.P., Chen L., Wang X.J., Jiang Q.H., Bei X.Y., Sun W.L., Xia S.J., Jiang J.T. (2017). Maternal exposure to di-n-butyl phthalate (DBP) induces renal fibrosis in adult rat offspring. Oncotarget.

[195] Fan Y., Qin Y., Chen M., Li X., Wang R., Huang Z., Xu Q., Yu M., Zhang Y., Han X. (2020). Prenatal low-dose DEHP exposure induces metabolic adaptation and obesity: Role of hepatic thiamine metabolism. J. Hazard. Mater..

[196] Yang J., Link C., Henderson Y.O., Bithi N., Hine C. (2021). Peripubertal Bisphenol A Exposure Imparts Detrimental Age-Related Changes in Body Composition, Cognition, and Hydrogen Sulfide Production Capacities. Antioxid. Redox Signal..

[197] Tain Y.L., Joles J.A. (2015). Reprogramming: A Preventive Strategy in Hypertension Focusing on the Kidney. Int. J. Mol. Sci..

[198] Hsu C.N., Hou C.Y., Chang-Chien G.P., Lin S., Tain Y.L. (2022). Dietary Supplementation with Cysteine during Pregnancy Rescues Maternal Chronic Kidney Disease-Induced Hypertension in Male Rat Offspring: The Impact of Hydrogen Sulfide and Microbiota-Derived Tryptophan Metabolites. Antioxidants.

[199] Luo Y., Tian Y., Zhao C. (2020). Taurine attenuates liver autophagy and injury of offspring in gestational diabetic mellitus rats. Life Sci..

[200] Cetin A.K., Buyukdere Y., Gulec A., Akyol A. (2023). Taurine supplementation reduces adiposity and hepatic lipid metabolic activity in adult offspring following maternal cafeteria diet. Nutr. Res..

[201] Roysommuti S., Lerdweeraphon W., Malila P., Jirakulsomchok D., Wyss J.M. (2009). Perinatal taurine alters arterial pressure control and renal function in adult offspring. Adv. Exp. Med. Biol..

[202] Horie R., Yamori Y., Nara Y., Sawamura M., Mano M. (1987). Effects of sulphur amino acids on the development of hypertension and atherosclerosis in stroke-prone spontaneously hypertensive rats. J. Hypertens. Suppl..

[203] Tai I.H., Sheen J.M., Lin Y.J., Yu H.R., Tiao M.M., Chen C.C., Huang L.T., Tain Y.L. (2016). Maternal N-acetylcysteine therapy regulates hydrogen sulfide-generating pathway and prevents programmed hypertension in male offspring exposed to prenatal dexamethasone and postnatal high-fat diet. Nitric Oxide.

[204] Tain Y.L., Lee C.T., Chan J.Y., Hsu C.N. (2016). Maternal melatonin or N-acetylcysteine therapy regulates hydrogen sulfide-generating pathway and renal transcriptome to prevent prenatal N(G)-Nitro-L-argininemethyl ester (L-NAME)-induced fetal programming of hypertension in adult male offspring. Am. J. Obstet. Gynecol..

[205] Tain Y.L., Hsu C.N., Lee C.T., Lin Y.J., Tsai C.C. (2016). N-Acetylcysteine Prevents Programmed Hypertension in Male Rat Offspring Born to Suramin-Treated Mothers. Biol. Reprod..

[206] Hsu C.N., Hou C.Y., Chang-Chien G.P., Lin S., Tain Y.L. (2020). Maternal N-Acetylcysteine Therapy Prevents Hypertension in Spontaneously Hypertensive Rat Offspring: Implications of Hydrogen Sulfide-Generating Pathway and Gut Microbiota. Antioxidants.

[207] Xiao D., Huang X., Li Y., Dasgupta C., Wang L., Zhang L. (2015). Antenatal Antioxidant Prevents Nicotine-Mediated Hypertensive Response in Rat Adult Offspring. Biol. Reprod..

[208] Michlin M., Argaev-Frenkel L., Weinstein-Fudim L., Ornoy A., Rosenzweig T. (2020). Maternal N-Acetyl Cysteine Intake Improved Glucose Tolerance in Obese Mice Offspring. Int. J. Mol. Sci..

[209] Yonatan E., Shukha O.N., Golani I., Abu-Ata S., Awad-Igbaria Y., Khatib N., Ginsberg Y., Palzur E., Beloosesky R., Shamir A. (2025). Maternal N-acetylcysteine supplementation in lactation ameliorates metabolic and cognitive deficits in adult offspring exposed to maternal obesity. Neuropharmacology.

[210] Guo Q., Feng X., Xue H., Teng X., Jin S., Duan X., Xiao L., Wu Y. (2017). Maternal Renovascular Hypertensive Rats Treatment with Hydrogen Sulfide Increased the Methylation of AT1b Gene in Offspring. Am. J. Hypertens..

[211] Feng X., Guo Q., Xue H., Duan X., Jin S., Wu Y. (2020). Hydrogen Sulfide Attenuated Angiotensin II-Induced Sympathetic Excitation in Offspring of Renovascular Hypertensive Rats. Front. Pharmacol..

[212] Tain Y.L., Hou C.Y., Chang-Chien G.P., Lin S., Hsu C.N. (2023). Protection by Means of Perinatal Oral Sodium Thiosulfate Administration against Offspring Hypertension in a Rat Model of Maternal Chronic Kidney Disease. Antioxidants.

[213] Tain Y.L., Hou C.Y., Chang-Chien G.P., Lin S., Hsu C.N. (2022). Perinatal Garlic Oil Supplementation Averts Rat Offspring Hypertension Programmed by Maternal Chronic Kidney Disease. Nutrients.

[214] Hsu C.N., Hou C.Y., Chang-Chien G.P., Lin S., Tain Y.L. (2021). Maternal Garlic Oil Supplementation Prevents High-Fat Diet-Induced Hypertension in Adult Rat Offspring: Implications of H_2_S-Generating Pathway in the Gut and Kidneys. Mol. Nutr. Food Res..

[215] Tain Y.L., Hou C.Y., Chang-Chien G.P., Lin S.F., Hsu C.N. (2024). Chondroitin Sulfate Ameliorates Hypertension in Male Offspring Rat Born to Mothers Fed an Adenine Diet. Antioxidants.

[216] Sengupta P. (2013). The Laboratory Rat: Relating Its Age with Human’s. Int. J. Prev. Med..

[217] Shibuya N., Kimura H. (2013). Production of hydrogen sulfide from D-cysteine and its therapeutic potential. Front. Endocrinol..

[218] Zhou L., Yan Z., Yang S., Lu G., Nie Y., Ren Y., Xue Y., Shi J.S., Xu Z.H., Geng Y. (2024). High methionine intake alters gut microbiota and lipid profile and leads to liver steatosis in mice. Food Funct..

[219] Chaturvedi P., Kamat P.K., Kalani A., Familtseva A., Tyagi S.C. (2016). High Methionine Diet Poses Cardiac Threat: A Molecular Insight. J. Cell Physiol..

[220] Powell C.R., Dillon K.M., Matson J.B. (2018). A review of hydrogen sulfide (H_2_S) donors: Chemistry and potential therapeutic applications. Biochem. Pharmacol..

[221] Wen Y.D., Wang H., Zhu Y.Z. (2018). The Drug Developments of Hydrogen Sulfide on Cardiovascular Disease. Cell. Longev..

[222] Wang K., Ahmad S., Cai M., Rennie J., Fujisawa T., Crispi F., Baily J., Miller M.R., Cudmore M., Hadoke P.W.F. (2013). Dysregulation of Hydrogen Sulfide Producing Enzyme Cystathionine γ-lyase Contributes to Maternal Hypertension and Placental Abnormalities in Preeclampsia. Circulation.

[223] Sharma D.K., Manral A., Saini V., Singh A., Srinivasan B., Tiwari M. (2012). Novel diallyldisulfide analogs ameliorate cardiovascular remodeling in rats with L-NAME-induced hypertension. Eur. J. Pharmacol..

[224] Nguyen I.T., Klooster A., Minnion M., Feelisch M., Verhaar M.C., Van Goor H., Joles J.A. (2020). Sodium thiosulfate improves renal function and oxygenation in L-NNA–induced hypertension in rats. Kidney Int..

[225] Snijder P.M., Frenay A.-R.S., Koning A.M., Bachtler M., Pasch A., Kwakernaak A.J., Berg E.V.D., Bos E.M., Hillebrands J.-L., Navis G. (2014). Sodium thiosulfate attenuates angiotensin II-induced hypertension, proteinuria and renal damage. Nitric Oxide.

[226] Piragine E., Citi V., Lawson K., Calderone V., Martelli A. (2022). Potential Effects of Natural H_2_S-Donors in Hypertension Management. Biomolecules.

[227] Imaizumi V.M., Laurindo L.F., Manzan B., Guiguer E.L., Oshiiwa M., Otoboni A.M.M.B., Araujo A.C., Tofano R.J., Barbalho S.M. (2023). Garlic: A systematic review of the effects on cardiovascular diseases. Crit. Rev. Food Sci. Nutr..

[228] Shouk R., Abdou A., Shetty K., Sarkar D., Eid A.H. (2014). Mechanisms underlying the antihypertensive effects of garlic bioactives. Nutr. Res..

[229] Ried K., Fakler P. (2014). Potential of garlic (*Allium sativum*) in lowering high blood pressure: Mechanisms of action and clinical relevance. Integr. Blood Press. Control.

[230] Ribeiro M., Alvarenga L., Cardozo L.F.M.F., Chermut T.R., Sequeira J., de Souza Gouveia Moreira L., Teixeira K.T.R., Shiels P.G., Stenvinkel P., Mafra D. (2021). From the distinctive smell to therapeutic effects: Garlic for cardiovascular, hepatic, gut, diabetes and chronic kidney disease. Clin. Nutr..

[231] Shen Q., Guo Y., Wang K., Zhang C., Ma Y. (2023). A Review of Chondroitin Sulfate’s Preparation, Properties, Functions, and Applications. Molecules.

[232] Pichette J., Fynn-Sackey N., Gagnon J. (2017). Hydrogen Sulfide and Sulfate Prebiotic Stimulates the Secretion of GLP-1 and Improves Glycemia in Male Mice. Endocrinology.

[233] Chen L., Gao Y., Zhao Y., Yang G., Wang C., Zhao Z., Li S. (2022). Chondroitin sulfate stimulates the secretion of H_2_S by Desulfovibrio to improve insulin sensitivity in NAFLD mice. Int. J. Biol. Macromol..

[234] Tomasova L., Konopelski P., Ufnal M. (2016). Gut Bacteria and Hydrogen Sulfide: The New Old Players in Circulatory System Homeostasis. Molecules.

[235] Dostal Webster A., Staley C., Hamilton M.J., Huang M., Fryxell K., Erickson R., Kabage A.J., Sadowsky M.J., Khoruts A. (2019). Influence of short-term changes in dietary sulfur on the relative abundances of intestinal sulfate-reducing bacteria. Gut Microbes.

[236] Hsu C.N., Tain Y.L. (2022). Chronic Kidney Disease and Gut Microbiota: What Is Their Connection in Early Life?. Int. J. Mol. Sci..

[237] Pandey K.R., Naik S.R., Vakil B.V. (2015). Probiotics, prebiotics and synbiotics—A review. J. Food Sci. Technol..

[238] Zółkiewicz J., Marzec A., Ruszczyn’ski M., Feleszko W. (2020). Postbiotics-A step beyond pre- and probiotics. Nutrients.

[239] Cutrell S., Alhomoud I.S., Mehta A., Talasaz A.H., Van Tassell B., Dixon D.L. (2023). ACE-Inhibitors in Hypertension: A Historical Perspective and Current Insights. Curr. Hypertens. Rep..

[240] Dutta S., Shah R.B., Singhal S., Dutta S.B., Bansal S., Sinha S., Haque M. (2023). Metformin: A Review of Potential Mechanism and Therapeutic Utility Beyond Diabetes. Drug Des. Dev. Ther..

[241] Tain Y.L., Hsu C.N. (2024). The Renin-Angiotensin System and Cardiovascular-Kidney-Metabolic Syndrome: Focus on Early-Life Programming. Int. J. Mol. Sci..

[242] Tain Y.L., Wu K.L.H., Lee W.C., Leu S., Chan J.Y.H. (2018). Prenatal Metformin Therapy Attenuates Hypertension of Developmental Origin in Male Adult Offspring Exposed to Maternal High-Fructose and Post-Weaning High-Fat Diets. Int. J. Mol. Sci..

[243] Li Z., Peng H., Huang Y., Lv B., Tang C., Du J., Yang J., Fu L., Jin H. (2024). Systematic analysis of the global characteristics and reciprocal effects of S-nitrosylation and S-persulfidation in the human proteome. Free Radic. Biol. Med..

[244] Liu H., Negoita F., Brook M., Sakamoto K., Morton N.M. (2024). Quantification of persulfidation on specific proteins: Are we nearly there yet?. Essays Biochem..

[245] Ali A., Wang Y., Wu L., Yang G. (2021). Gasotransmitter signaling in energy homeostasis and metabolic disorders. Free Radic. Res..

[246] Hendriks K.D., Maassen H., van Dijk P.R., Henning R.H., van Goor H., Hillebrands J.L. (2019). Gasotransmitters in health and disease: A mitochondria-centered view. Curr. Opin. Pharmacol..

[247] Huang Y.Q., Jin H.F., Zhang H., Tang C.S., Du J.B. (2021). Interaction among Hydrogen Sulfide and Other Gasotransmitters in Mammalian Physiology and Pathophysiology. Adv. Exp. Med. Biol..

[248] Hsu C.N., Tain Y.L. (2021). Gasotransmitters for the Therapeutic Prevention of Hypertension and Kidney Disease. Int. J. Mol. Sci..

[249] Tsakiridis I., Kasapidou E., Dagklis T., Leonida I., Leonida C., Bakaloudi D.R., Chourdakis M. (2020). Nutrition in Pregnancy: A Comparative Review of Major Guidelines. Obstet. Gynecol. Surv..

[250] Tain Y.L., Hsu C.N. (2024). Maternal Dietary Strategies for Improving Offspring Cardiovascular-Kidney-Metabolic Health: A Scoping Review. Int. J. Mol. Sci..

[251] Tain Y.L. (2025). Advocacy for DOHaD research optimizing child kidney health. Pediatr. Neonatol..

[252] McKerracher L., Moffat T., Barker M., Williams D., Sloboda D.M. (2019). Translating the Developmental Origins of Health and Disease concept to improve the nutritional environment for our next generations: A call for a reflexive, positive, multi-level approach. J. Dev. Orig. Health Dis..

